# Global long terminal repeat activation participates in establishing the unique gene expression programme of classical Hodgkin lymphoma

**DOI:** 10.1038/s41375-018-0311-x

**Published:** 2018-12-13

**Authors:** Benjamin Edginton-White, Pierre Cauchy, Salam A. Assi, Sylvia Hartmann, Arthur G. Riggs, Stephan Mathas, Peter N. Cockerill, Constanze Bonifer

**Affiliations:** 1 Institute for Cancer and Genomic Sciences, University of Birmingham, College of Medical and Dental Sciences, Birmingham, B152TT UK; 2 Senckenberg Institute of Pathology, University Hospital, 60590 Frankfurt, Germany; 3 Beckman Research Institute of City of Hope Medical Center, Duarte, CA 91010 USA; 4 Max-Delbrück-Center for Molecular Medicine, 13125 Berlin, Germany; 5 Hematology, Oncology, and Tumor Immunology, Charité – Universitätsmedizin Berlin, 12200 Berlin, Germany; 6 Present Address: Department of Cellular and Molecular Immunology, Max Planck Institute for Immunobiology and Epigenetics, 79108 Freiburg, Germany

**Keywords:** Genetics research, Hodgkin lymphoma

## Abstract

Long terminal repeat (LTR) elements are wide-spread in the human genome and have the potential to act as promoters and enhancers. Their expression is therefore under tight epigenetic control. We previously reported in classical Hodgkin Lymphoma (cHL) that a member of the *THE1B* class of LTR elements acted as a promoter for the proto-oncogene and growth factor receptor gene *CSF1R* and that expression of this gene is required for cHL tumour survival. However, to which extent and how such elements participate in globally shaping the unique cHL gene expression programme is unknown. To address this question we mapped the genome-wide activation of *THE1-*LTRs in cHL cells using a targeted next generation sequencing approach (RACE-Seq). Integration of these data with global gene expression data from cHL and control B cell lines showed a unique pattern of LTR activation impacting on gene expression, including genes associated with the cHL phenotype. We also show that global LTR activation is induced by strong inflammatory stimuli. Together these results demonstrate that LTR activation provides an additional layer of gene deregulation in classical Hodgkin lymphoma and highlight the potential impact of genome-wide LTR activation in other inflammatory diseases.

## Introduction

Human endogenous retroviruses (HERVs) account for 8% of the human genome originating from ancient retroviral germ line infections arising over 35 million years ago and persisting throughout mammalian evolution [1, 2]. Mammalian apparent long terminal repeat (LTR) retrotransposons (MaLR) are the largest HERV class (48%) [2] and consist mainly of solitary LTRs spread throughout the genome [1]. LTRs need to be strictly regulated due to their potential to act as RNA-polymerase II-dependent promoters and enhancers [3]. LTR activation in disease can lead to dysregulation of oncogene expression [4]. We showed this function of LTRs for the first time in malignant Hodgkin–/Reed–Sternberg (HRS) cells in classical Hodgkin lymphoma (cHL) [5] which exhibit a highly dysregulated inflammatory gene expression programme unrelated to that of normal B-cells [6]. Due to the loss of B-cell receptor (BCR) expression, HRS cells developed alternative survival mechanisms, one of which is the co-expression of *CSF1* and *CSF1R* [5]. We showed that *CSF1R* expression is driven by an aberrantly activated upstream *THE1B* LTR which belongs to the MalR family of repeat elements. Activation of this LTR was associated with a down-regulation of expression of the co-repressor ETO2 (*CBFA2T3)* and NF-κB-activation [5]. Using 3′ rapid amplification of cDNA ends (RACE) we also showed that *THE1B* family LTR activation in HRS cells was wide-spread. However, which of these *THE1B* LTRs served as promoters in HL, and the impact of *THE1-LTR* activation on global gene expression was not investigated.

To answer this question, we designed a genome-wide targeted sequencing assay to further investigate the activation of *THE1*-LTRs in HRS cell lines, primary cells and control B-cell lines. We find unique patterns of HRS cell-specific activation of *THE1B*-LTR promoters at genes associated with the cHL phenotype and show that genome-wide LTR activation is triggered by the activation of NF-κB and broader inflammatory stimuli. Our results highlight the potential for *THE1B-*LTRs to have widespread influence on gene regulation in cHL and other inflammatory diseases.

## Methods

### Cell culture

cHL (L428, L1236 and KM-H2), Burkitt’s lymphoma (Namalwa) and B-cell precursor leukaemia (Reh) cell lines were cultured as previously described [5]. The 293T cell line was cultured in DMEM with 10% FCS, 2 mM Glutamine, plus P/S. All cultures were incubated at 37 °C in a humidified incubator with 5% CO_2_.

### Gene expression analysis

RNA was isolated using a Nucleospin RNA extraction column with on-column DNase treatment (Machery Nagel, France). RT-qPCR was performed using Sybr^®^ green master mix (Sigma) and was run on an Applied Biosystems StepOne Plus RT PCR system with the default PCR programme (Primer sequences, see supplements). All expression values were normalised to *GAPDH* expression and a Student's *t*-test was used to test statistical significance for data with a normal distribution.

RNA sequencing libraries were produced in duplicate using the TruSeq Stranded Total RNA Library Prep Kit with Ribo-Zero Human/Mouse/Rat (Illumina). Next Generation Sequencing (NGS) was carried out using Illumina HiSeq 2500 and NextSeq 500 instruments.

### RACE-Seq

RACE was carried out based on the ExactSTART™ Eukaryotic mRNA 5′-RACE and 3′-RACE Kit (Epicentre) and supplied protocol. Due to discontinuation of the Tobacco Acid Pyrophosphatase enzyme some modifications were made (see Supplementary materials). A biotinylated primer complementary to a highly conserved transcribed region of the *THE1B* LTR was used to synthesise RACE fragments. Fragments were then pulled down using magnetic streptavidin beads, amplified and tagged with indexed Illumina sequencing adaptors to allow for direct next generation sequencing (NGS).

### Western blotting

Total protein was extracted using RIPA buffer with protease inhibitors added (1:100). Nuclear protein extraction was carried out using the Active Motif Nuclear Extraction Kit. Western blotting was performed by blotting Mini-PROTEAN TGX 5–20% gradient gels (Bio-Rad) on nitrocellulose membranes using the Trans-Blot Turbo system (Bio-Rad). Blocking was carried out using 5% milk in TBST. Antibodies are listed in Supplementary Materials.

### Phorbol myristate acetate (PMA) treatment

PMA treatment was at 2 ng/ml media at a cell density of 1 × 10^6^/ml.

### RNA interference

siRNA knockdown was performed by re-suspending L1236 and KM-H2 cells at 1 × 10^7^ cells/ml in 700 µl Opti-MEM medium (Sigma) and mixed with siRNA to a final concentration of 200 nM in a 0.4 mm electroporation cuvette and electroporated using a Gene-Pulser X-cell (Bio-Rad) (L1236–960 µF, 0.18 kV and KM-H2–50 μF, 0.5 kV).

### IκKβ(EE) cloning

IκKβ(EE) DNA was excised from a plasmid described in ref. [5] and transferred to the PCW57.1 vector (Addgene plasmid # 41393) using Gateway cloning. Viral particles were produced using Hek293T cells as described [7]. Reh cells were transduced by spin infection (1500×*g*, 2 h) and puromycin (1 μg/ml) selected for 1 week. Following doxycycline induction (2 μg/ml) for 48 h, cells were purified by fluorescence-activated cell sorting for GFP labelling and immediately used for experiments.

### Laser capture microdissection and low input RNA-Seq library preparation

Frozen tumour tissue was fixed for 10 min with 100% ethanol and air dried. Following fixation 200–400  HRS or surrounding bystander cells were excised using a Zeiss PALM LCM system and catapulted onto adhesive caps (415190-9191-000—Zeiss). Cell lysis, RNA extraction and purification were performed using the absolutely RNA Nanoprep Kit (400753—Agilent). RNA-Seq libraries were produced using the SMARTer Stranded Total RNA-Seq Kit v2—Pico input Mammalian (Takara Bio). The libraries were used for both rtPCR as previously described and were sequenced on an Illumina NextSeq 500 at the Genomics Birmingham facility.

### ChIP-Seq

ChIP was performed as previously described [8] with slight modifications, i.e. using 5 × 10^6^ cells, 15 µl Protein-G Dynabeads (Invitrogen), 2 µg H3K4me3 millipore 04745 antibody, and using phenol–chloroform DNA extraction. Detailed methods are available in Supplementary Methods. Libraries were generated using the Kapa Hyperprep Kit (Kapa Biosystems) according to manufacturer’s instructions and sequenced using an Illumina HiSeq 2500 sequencer.

### Data analysis

Full details of bioinformatics analysis can be found in the Supplementary Materials.

## Results

### cHL cell lines display a global activation of *MalR* long terminal repeat elements

**Fig. 1:**
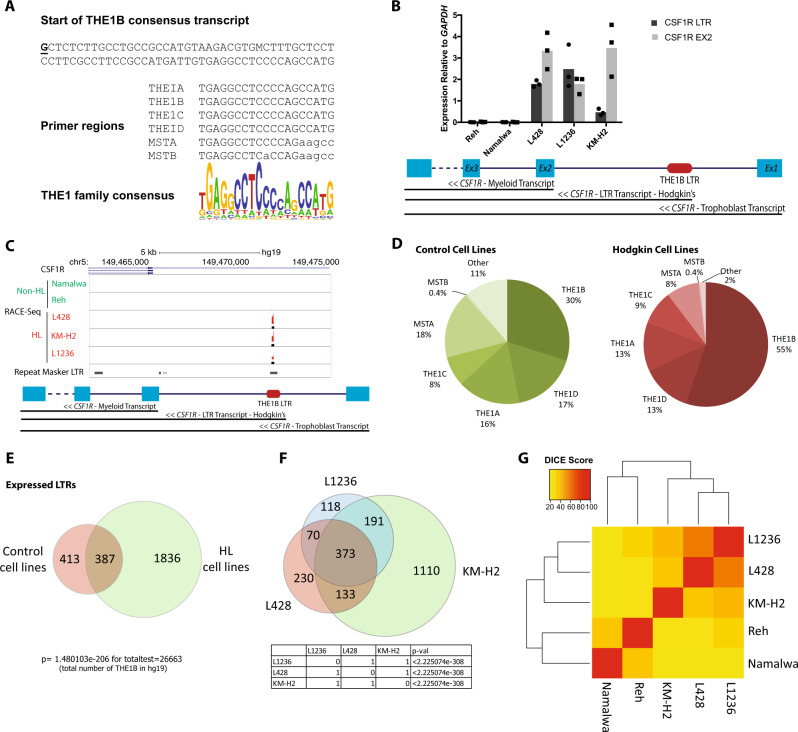
HRS cell lines display a global activation of long terminal repeat elements. **a** Sequence of the 82 base 5′ terminal region of the consensus THE1B transcript, plus the regions corresponding to the 5′ RACE primer region in the MaLR family member amplified by the primer, together with the overall THE1 consensus sequence. **b** RT-qPCR gene expression analysis measuring LTR-driven *CSF1R* expression (normalised to *GAPDH* expression). *n* = 3. Up-regulation in HRS cell lines *P* < 0.05, paired Student's *t*-test. **c** Alignment of RACE-Seq fragments to the human genome (hg19). Regions identified as RACE-Seq peaks are shown as black bars under fragment alignments. **d** Annotations of RACE-Seq peaks with the Repeat Masker hg19 repeat family annotation. **e** Overlap of active LTRs detected by RACE-Seq in 3 HRS cell lines (L428, L1236, and KM-H2) (green) and two control cell lines (Reh and Namalwa) (red). **f** Overlap of active LTRs detected by RACE-Seq in three cHL cell lines. **g** Clustering based on DICE index scores calculated for each pair of cell lines based on active LTR presence identified by RACE-Seq

To identify the global pattern of LTR-driven transcripts in HRS cells we designed a genome-wide 5′ RACE-Seq screen using a primer matching a highly conserved, transcribed LTR region. This sequence is shared between *THE1A, THE1B*, *THE1C*, and *THE1D* LTRs, and also amplifies *MSTA* and *MSTB* LTRs (Fig. 1a and S1A). RACE products from L428, L1236 and KM-H2 cHL cell lines expressing the LTR-driven CSF1R, and Reh and Namalwa cell lines representing non-Hodgkin B cells as controls (Fig. 1b) were analysed by NGS. In agreement with our previous work [5], 5′ RACE-Seq showed a peak corresponding to the *CSF1R*-LTR in the genome of all cHL cell lines but not in controls (Fig. 1c). The consensus *THE1B* primer (Fig. 1a) amplified a broad library of related LTR classes, including *THE1B, THE1D, THE1C, THE1A*, and *MSTA* (Fig. 1d and S1B). As expected, cHL cell lines displayed a bias towards *THE1B* activation (over 50% of the detected LTRs) when compared to the balance of LTR classes in the genome and to those active in the control cell lines (Fig. 1d and S1B). Only 10% of the LTRs perfectly aligned to the primer sequence, with amplified LTRs having up to eight mismatches (Fig. S1C). Due to the high proportion of mismatches, only 7–30% of peaks were shared between all three RACE-Seq replicates in each cell line (Fig. S1D). This variation was likely to be due to variations in the crucial first extension step, we therefore used a conservative approach by carrying out all further analysis on LTRs identified in at least two out of the three replicates.

To identify a cHL cell-specific pattern of LTR activation, we overlapped merged RACE-Seq peaks from all three cHL cell lines with the merged peaks from the two control cell lines (Fig. 1e). This analysis highlighted 1836 cHL cell-specific active LTRs and 413 LTRs specific to the control cell lines. Additionally, we identified 373 activated LTRs shared between cHL cell lines (of which 224 are unique to HL) but also LTRs unique for each cell line (Fig. 1f), a variation that was significant based on hypergeometric analysis (*p* < 0.01). To find similarities between the cHL cell lines, we performed a clustering analysis based on a DICE index score which was calculated for each pair (Fig. 1g). This analysis showed that the cHL cell line LTR-profiles clustered together, away from the controls.

### LTR activation contributes to global deregulation of gene expression in cHL cells

**Fig. 2:**
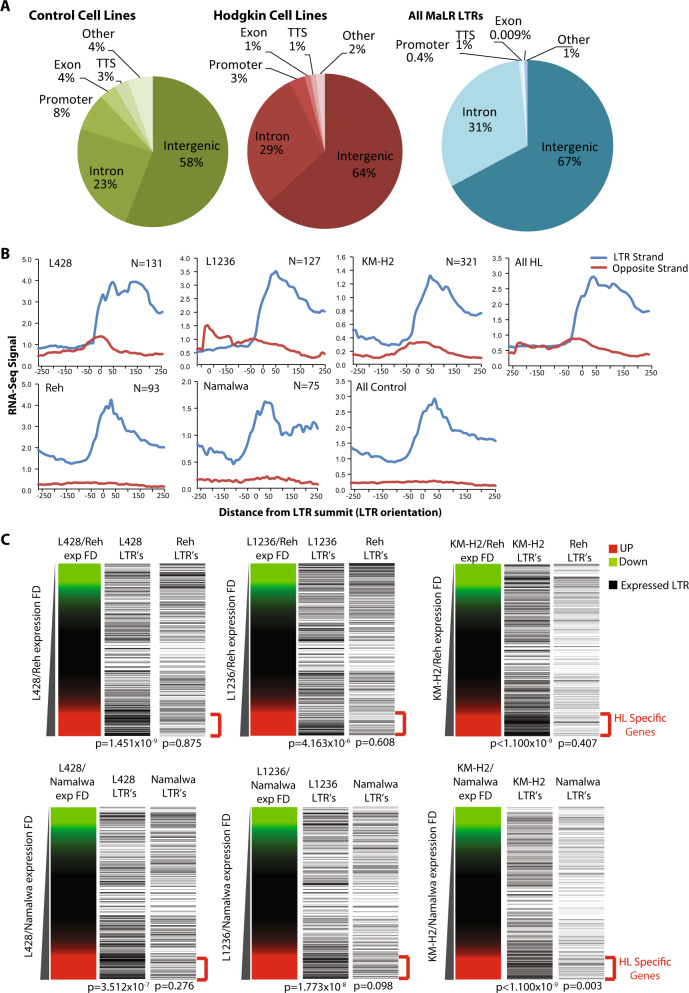
LTR activation contributes to global deregulation of gene expression in cHL cell lines. **a** Annotation of expressed LTRs identified by RACE-Seq to their respective genomic regions and annotation of all RepeatMasker MaLR family LTRs for comparison. **b** Average profiles produced using RNA-Seq data centred on active LTRs identified by THE1B RACE-Seq and that were selected as upstream of genes (i.e. as having the nearest downstream gene on the same strand, and outside of promoter regions defined as 1 kb to +100 bp from the TSS), orientated based on LTR strand. ‘N’ represents number of LTRs applying to these constraints. **c** Plot of fold-change of gene expression obtained by RNA-Seq for each of the HRS cell lines over each of the control cell lines. Active LTRs close to these genes were plotted based on the gene expression fold-change axis. Significance of LTRs associated with upregulated genes tested using a hypergeometric test

The majority of transcribed LTRs were located in intra-genic and inter-genic regions (Figs. 2a and S2F) with 1–3% located at the transcription start site (TSS) of expressed genes (Fig. S2F). To investigate whether LTRs could act as promoters, we determined the gene expression profiles of the cHL cell lines using RNA-Seq (Fig. S2A, B) and plotted the RNA-Seq signal around active LTRs outside of promoter regions (defined as 1 kb to +100 bp from the TSS), using strand information inferred from Repeat Masker annotation (Fig. 2b). The previously mapped HL-specific LTR-driven transcript of *CSF1R* [9] is shown as control (Fig. S2C). An RNA-Seq signal was observed at active LTRs and stretched downstream on the sense strand. Only a small proportion of LTR-driven anti-sense transcripts were observed. Further to this analysis, we performed H3K4me3 ChIP-Seq in L428 cells and showed an enrichment of H3K4me3 at active LTRs (Figs. S2D and E) supporting the idea that these LTRs act as promoters.

HRS cells show a vastly different gene expression pattern to B lineage cells as shown by clustering analysis of RNA-Seq data (Fig. S2G). To examine whether active LTRs increased expression of their nearest genes, we clustered correlations of gene expression data for those genes closest to active LTRs. HRS and control cells clustered separately based solely on the expression of LTR-associated genes (Fig. S2G and S2H, for full list of RACE peaks and closest gene see Supplementary dataset 1). To link the active LTRs with cell-type-specific gene expression, we plotted the expression of LTR-associated genes in relation to the fold difference between HRS and control cell lines (Fig. 2c). This analysis showed a significant proportion of active LTRs close to the genes up-regulated in each of the HRS cell lines (red) compared to the control cell lines (green).

To understand how active LTRs influence expression of individual genes and contribute to the HRS cell phenotype, we manually screened active LTRs to examine surrounding changes in RNA expression. *THE1* LTRs produced four types of transcripts: (1) intergenic, acting as an upstream promoter up-regulating expression of a gene, (2) intragenic, with the LTR promoter producing a new shorter isoform of a gene, (3) anti-sense, leading to down-regulation of gene expression, or (4) intergenic as previously un-annotated long non-coding RNA (lncRNAs). Examples of each type of transcript are shown in Fig S3 and were validated by qPCR (Fig. S4A-G). Taken together, our data show a genome-wide transcriptional activation of *THE1* LTR members in cHL cells with a number of these elements altering the transcriptional output of their associated genes.

### LTR activation drives the expression of genes associated with cHL pathology

**Fig. 3:**
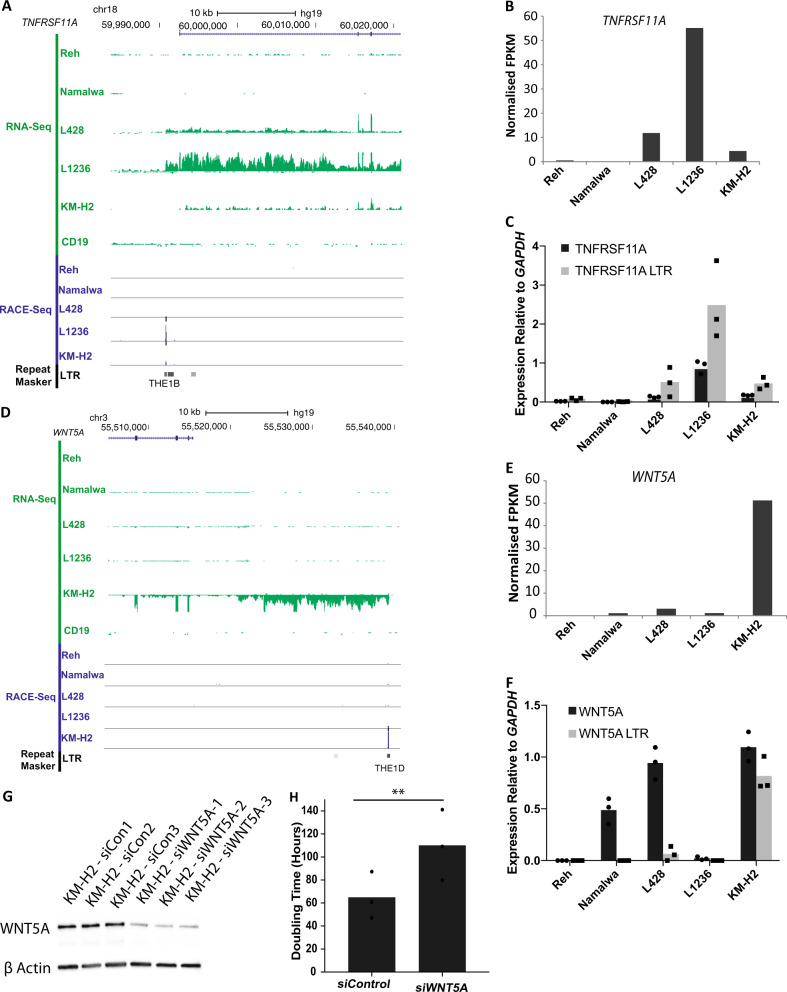
*TNFRSF11A* and *WNT5A* are expressed from active THE1B and THE1D LTRs. **a** UCSC genome browser screenshot showing a THE1B LTR acting as a promoter for the *TNFRSF11A* gene in L1236 and L428 cHL cell lines but not in primary CD19^+^ B cells [27]. **b** Normalised RNA-Seq FPKM values showing expression of *TNFRSF11A*. **c** qPCR gene expression analysis showing expression of a transcript between exon 2 and 3 and also between the upstream LTR and exon 2. Three biological replicates (*p* < 0.05 L1236 vs. control cell lines, paired Student's *t*-test). **d** UCSC genome browser screenshot showing an LTR identified by RACE-Seq in the KM-H2 cell line which produces a transcript of the *WNT5A* gene, shown by RNA-Seq read alignment. Compared to Primary CD19^+^ B cell RNA-Seq [27]. **e** Normalised RNA-Seq FPKM values showing expression of *WNT5A*. **f** qPCR gene expression analysis showing expression of a transcript between exon 2 and 3 and also between the upstream LTR and exon 2 of *WNT5A*. Three biological replicates (*p* < 0.01 KM-H2 vs. control cell lines, paired Student's *t*-test). **g** WNT5A protein measured by western blot following siRNA knockdown compared to non-targeting control. **h** Cell doubling time following siRNA knockdown of WNT5A. Three biological replicates (*p* < 0.01, paired Student's *t-*test)

We next determined whether any of the newly discovered LTR-driven genes were associated with the HRS cell phenotype. The *TNFRSF11A* (tumour necrosis factor receptor superfamily member 11a) gene has been previously shown to be upregulated in cHL [10]. Our data showed an active *THE1B* LTR 1.2 kb upstream of *TNFRSF11A* producing a transcript in the L1236 cell line and also a weak transcript in the L428 cell line (Fig. 3a), leading to an increase in expression (Fig. 3b, c). To determine whether the LTR drives a full length transcript, we knocked down the *TNFRSF11A* mRNA with an siRNA designed against exon 7 and found that this also reduced the LTR-driven transcript (Fig. S5A and B). Interestingly, we identified a secondary mechanism of control for LTR-driven *TNFRSF11A* expression in the L1236 cell line. This specific LTR contains a NFI transcription factor-binding site (Fig. S6A). Nuclear Factor I X (NFIX) is highly expressed in L1236 cells, and following siRNA knockdown the expression of *TNFRSF11A* and its LTR transcript were significantly reduced (Figs. S6B and C).

A *THE1D* LTR 20 kb upstream of the *WNT5A* gene drives a transcript in the KM-H2 cell line (Fig. 3d–f) producing both a spliced and a lncRNA (annotated by GENCODE as RP11-875H7.5) (Fig. 3d and S5C). siRNA knockdown of *WNT5A* (Fig. 3g) leads to a significant increase in cell doubling time (Fig. 3h), demonstrating a functional role for another active LTR in determining the HRS cell phenotype.

### LTR-driven transcripts can be detected in primary HRS cells

**Fig. 4:**
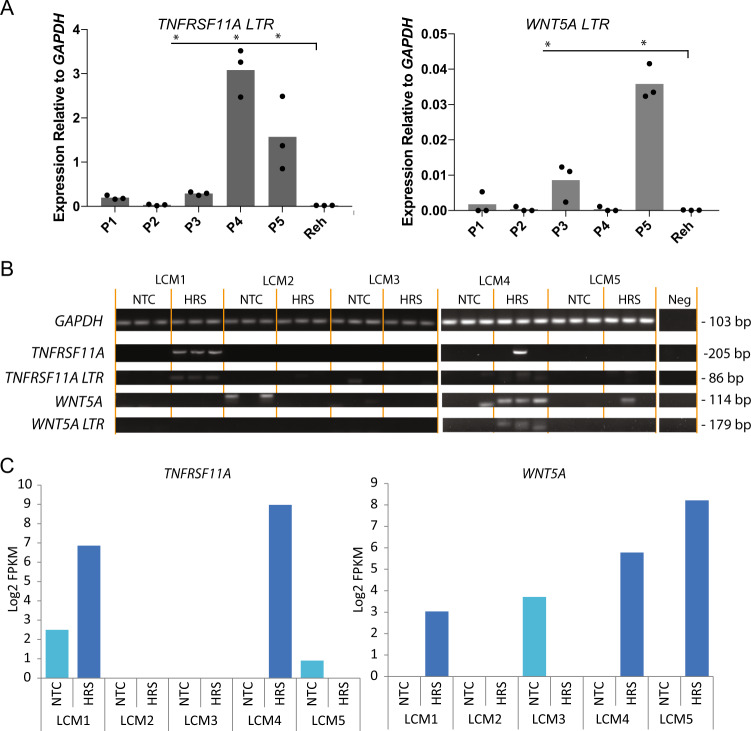
*TNFRSF11A* and *WNT5A LTR* transcripts can be detected in primary cHL tumour tissue and HRS cells. **a** qPCR measuring *TNFRSF11A-LTR and  WNT5A-LTR* transcript expression performed on RNA extracted from frozen cHL tumour samples (P1–P5) and Reh cells (negative control). Three technical replicates (*p < 0.05 paired Student's *t*-test). **b** RT-PCR performed on cDNA from laser capture micro-dissected (LCM) cells from five tumour samples (LCM1–LCM5) comparing HRS cells to bystander cells (NTC), a mixed infiltrate of immune cells. **c** Gene expression of *TNFRSF11A* and *WNT5A* measured by RNA-Seq in the LCM tumour cells

To examine whether the LTR-driven pattern of gene activation identified in the cell lines was also present in primary cells, we analysed RNA from frozen HL tumours. The *TNFRSF11A* LTR transcript was significantly up-regulated in three out of the five patient samples, correlating with up-regulation of the gene in two out of the three cell lines (Fig. 4a and S7A). The *WNT5A* LTR-driven transcript was expressed in two out of the five samples which again correlated with *WNT5A* up-regulation (Fig. 4a and S7A). A HL tumour can be composed of as little as 1% HRS cells [11]. We therefore analysed RNA from laser capture micro-dissected (LCM) HRS cells and surrounding bystander cells from five different tumours. RT-PCR showed the expression of a 86 bp band indicative for the *TNFRSF11A* LTR transcript in one sample and the 179 bp band indicating the *WNT5A* LTR transcript in another (Fig. 4b). We further characterised LCM cells by RNA-Seq and confirmed that isolated HRS cells expressed common up-regulated genes and the HRS-specific up-regulated gene signature identified in [12] (Figs. S7 B, C and D). All primary HRS cells specifically expressed *TNFRSF8* (CD30) and most HRS and bystander cells expressed CD40 (Figs. S7E and F), highlighting the quality of our data However, only a subset of tumours expressed LTR-driven *TNFRSF11A* and *WNT5A* transcripts (Fig. 4c). Further to our finding that NFIX regulates the *TNFRSF11A*-LTR the expression of NFIX in the particular LCM sample correlated with the presence of a *TNFRSF11A-*LTR transcript (Fig. S7G).

### *THE1* LTRs are activated by inflammatory stimuli

Our previous analyses [5] identified a NF-κB-binding motif in the *CSF1R-*LTR (Fig. S8A) and demonstrated that NF-κB signalling plus knock-down of *CBFA2T3* (ETO2) activated this element in non-Hodgkin cells. To determine whether constitutive NF-κB signalling alone could induce global *THE1B* LTR activation, we transduced the non-Hodgkin Reh cell line with a doxycycline inducible activating *IKKβ* (IKKβ(EE)) (Figs. S8B, C and D) [13] followed by RACE-Seq experiments. Results were filtered for those present in at least two replicates (Fig. S8E). NF-κB activation resulted in activation of 620 additional LTRs as compared to un-induced and GFP-only induced cells (Fig. S8F). 161 of the NF-κB-driven LTRs were also expressed in HRS cells compared with 16 LTRs from un-induced cells (Fig. S8G). However, a further 531 LTRs were being activated specifically in Reh cells (Fig. S8G), implying that *THE1* LTR activation across the genome is controlled by cell-type-specific regulatory mechanisms such as CBFA2T3 expression.

**Fig. 5:**
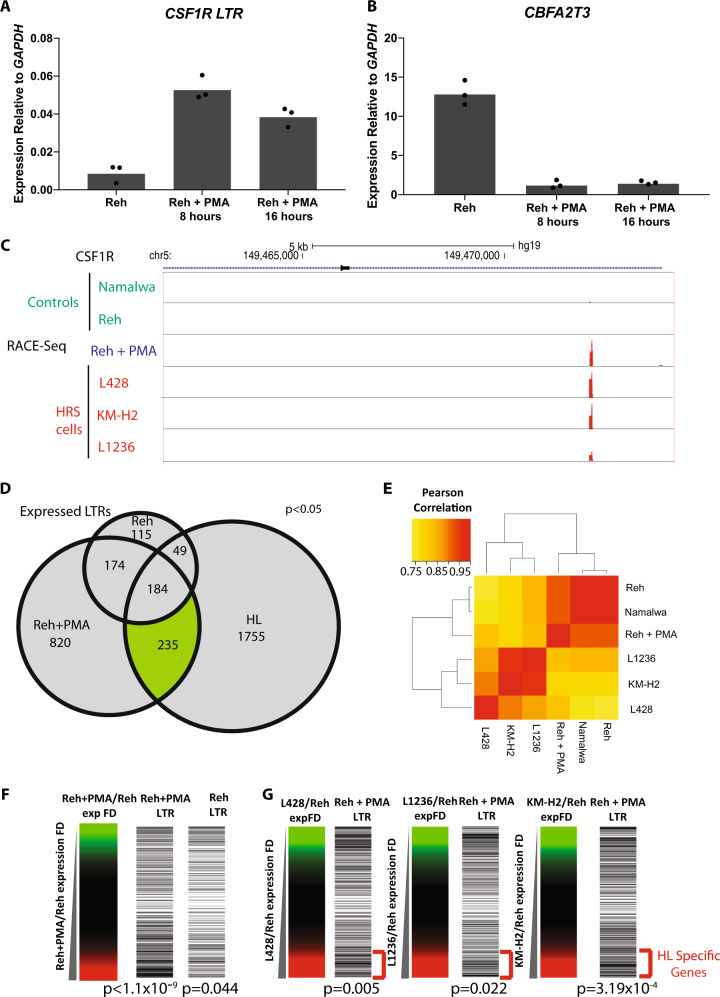
Treatment of the Reh cell line with PMA induces global *THE1B LTR* activation. **a** Treatment of Reh cells with 2 ng/ml PMA for 8 or 16 h followed by measurement of *CSF1R-LTR* expression by qPCR. Three biological replicates. (*p* < 0.05 PMA treated vs. untreated, paired Student's *t*-test) **b** *CBFA2T3* gene expression measured by qPCR following treatment of Reh cells with 2 ng/ml PMA for 8 or 16 h. Three biological replicates (*p* < 0.05 PMA treated vs. untreated, paired Student's *t*-test). **c** UCSC genome browser screenshot showing alignment of RACE-Seq reads to the *CSF1R-LTR* following PMA treatment of Reh cells. **d** Overlap of RACE-Seq LTR peaks from Reh cells±PMA treatment with merged peaks from the 3 HRS cell lines. **e** Hierarchical unsupervised clustering of Pearson correlation of gene expression patterns. **f** Fold-change of gene expression before and after PMA treatment with the presence of active LTRs close to these genes plotted based on the gene expression fold-change axis. **g** Fold-change of gene expression obtained by RNA-Seq for each HRS cell line over Reh with the presence of active PMA induced LTRs close to these genes plotted based on the gene expression fold-change axis. Significance of LTRs associated with upregulated genes tested using a hypergeometric test

HRS cells display a strong inflammatory gene expression signature [6] as seen in the KEGG pathway analysis based on our RNA-Seq data (Fig. S9A). To study the impact of a broader inflammatory stimulus on LTR activation, Reh cells were treated with phorbol-12-myristate 13-acetate (PMA), a potent activator of protein kinase C (PKC) for 8 or 16 h [14]. PKC activation drives multiple pathways linked to inflammation and tumour growth, many of which are also upregulated in HRS cells [15]. The *CSF1R* LTR was up-regulated at both time points with a peak at 8 h (Fig. 5a) whereby cells stopped growing in the presence of PMA (Fig. S9B). Crucially, PMA treatment also led to the rapid loss of *CBFA2T3* expression (Fig. 5b). RNA-Seq analysis showed the expected activation of inflammatory response genes (Fig. S9D and E) with a number of them overlapping with those active in HRS cells, such as *CCL4* and *RELB* (Fig. S9F and G). Triplicate RACE-Seq data obtained after 8 h of treatment were again filtered for high confidence peaks (Fig. S9C) and confirmed activation of the *CSF1R* LTR (Fig. 5c). Expression of 235 cHL cell-specific LTRs was induced by PMA treatment but a further additional 820 LTRs were also activated, again demonstrating that activation was cell-type specific (Fig. 5d). Integration of RACE-Seq with RNA-Seq data demonstrated that total gene expression patterns of the different cell lines clustered based on the different cell types (Fig. 5e). However, the analysis of LTR-associated genes showed that gene expression patterns in Reh+PMA cells started to correlate closer with HRS cells, with genes both being up-regulated and down-regulated (brackets in Fig. S9F). In addition, we observed an enrichment of up-regulated genes near PMA-activated LTRs (Fig.5f). To establish whether the genes upregulated by PMA-inducible LTRs were also up-regulated by LTRs in HRS cell lines, we ranked the gene expression fold change of several HRS cell lines over Reh and aligned ranked genes with the presence of neighbouring LTRs (Fig. 5g). This analysis showed that PMA treatment induced expression of LTRs associated with up-regulated genes in cHL.

## Discussion

### Global *THE1* long terminal repeat element activation contributes to deregulation of gene expression in classical Hodgkin lymphoma

In our previous work, we observed wide-spread activation of LTR-associated transcripts in cHL [5], suggesting that LTR activation could be playing a wider role in driving cHL-specific gene expression. Here we used a targeted approach to analyse the global activation of the *THE1* class of LTRs in cHL cell lines and primary material. In concordance with other studies (reviewed by Babaian and Mager [4]) we identified four types of LTR-driven transcripts and showed the up-regulation of cHL-specific genes near active LTRs, thus contributing to the overall expression pattern of each cell line. However, while there is a shared cHL pattern of LTR activation, we also observed cellular heterogeneity within the different cell lines, as well as in the tumour cell population. The reason for this heterogeneity is unclear, but may originate from cell-type-specific LTR activation as seen in our stimulation experiments. It should also be noted that LTRs could act as enhancers of their nearest genes, further deregulating gene expression from a distance, contributing to the correlation seen in Fig. 2c.

### HRS-specific genes transcribed from activated LTRs play a role in determining the cHL phenotype

We identified three protein-coding genes transcribed from an LTR promoter in cHL; *NLRP1*, *TNFRSF11A*, and *WNT5A*. We focused on *TNFRSF11A* and *WNT5A* as their upregulation has previously been reported in cHL [6, 10, 16]. *TNFRSF11A* is up-regulated in an average of 75% of HRS cells from primary tumour samples [6, 10]. Our work agrees with this finding and identifies an active *THE1B LTR* as the molecular mechanism of up-regulation. TNFRSF11A is a member of the TNF receptor family and binds TNFSF11A (RANKL) [17] which up-regulates signalling in a number of pathways, including NF-κB and JNK [17, 18]. In HRS cells, stimulation of TNFRSF11A signalling by TNFSF11A increases NF-κB-activation [10], leading to an increase in inflammatory signalling, thus potentially forming part of an autoregulatory loop through LTR activation.

The aberrant *WNT5A* transcript in HRS cells also originates from an upstream *THE1D LTR*. WNT ligands are involved in development and many cellular processes [19]WNT5A activates a β-catenin independent pathway driving metastasis of gastric, brain, colon, and breast cancer [20–22]. The up-regulation of *WNT5A* in HRS cells within a proportion of tumours has been noted [12, 16, 23] and a recent study implicated WNT5A in cell migration in HL [16]. WNT5A interacts with the Fzd5 receptor, leading to activation of RHOA which promotes motility in HRS cells [23]. Our knock-down experiments indicate that the LTR-driven transcript WNT5A may play a role in growth rate regulation in a subset of cHL.

### THE1 LTRs respond to inflammatory stimuli

The *CSF1R* LTR could be activated in non-Hodgkin cell by a combination of NF-κB activation and knockdown of the transcriptional repressor CBFA2T3 [4]. Both these conditions mirror the status of HRS cells in which NF-kB is constitutively activated and CBFA2T3 expression is frequently lost [4]. Here we show that induction of NF-κB activity alone resulted in the expression of a large number of LTRs. However, only a small proportion of these LTRs were also expressed in the HRS cell lines and the *CSF1R* LTR was not activated. This finding highlights the multiple levels of cell-type-specific control of LTR activation which is further supported by our observation that the *TNFRSF11A* LTR requires cell-type-specific NFIX expression to be transcriptionally active.

PMA activates a variety of different signalling pathways terminating at different inducible transcription factors [14] and induces expression of many more THE1 LTRs than NF-κB alone. The induction of ERV-expression by treatment with PMA has been shown previously, but not on a genome-wide scale, and the mechanism of induction was unclear [24–26]. Combined with the evidence for LTR-driven gene expression in this study, our findings have significant implications for control of gene expression in any tissue or disease with a high level of inflammation.

Our data support the idea that an initial inflammatory stimulus could act to drive widespread changes in gene expression as a result of LTR activation. The presence of *THE1* transcripts may be a marker for such a chronic condition. Transcribed LTRs pose a significant threat to correct regulation of gene expression and as shown here, contribute to the establishment of a tumour-specific transcriptional network potentially driving survival and growth of HRS cells. Global LTR activation through chronic inflammatory signalling therefore has broad implications for gene regulation in many inflammatory diseases.

## Supplementary information

Supplementary figures and methods

Supplementary dataset 1

## Data Availability

All raw and processed high-throughput sequencing data generated in this study were uploaded to the Gene Expression Omnibus (GEO) under accession numbers GSE120328 (primary tumour RNA-Seq), GSE120329 (RACE-Seq), GSE120538 (ChIP-Seq) and GSE120330 (RNA-Seq in cell lines).
