# Supplementary material for: Global long terminal repeat activation participates in establishing the unique gene expression programme of classical Hodgkin lymphoma
Source: Leukemia. 2018 Dec 13;33(6):1463–74. doi: 10.1038/s41375-018-0311-x (PMC6558280; doi:10.1038/s41375-018-0311-x)
Supplement: Supplementary file 1 — Supplementary figures and methods [file 41375_2018_311_MOESM1_ESM.pdf]

Global Long Terminal Repeat activation participates in establishing the unique gene expression program of classical Hodgkin Lymphoma.

Benjamin Edginton-White[1], Pierre Cauchy[1,*], Salam. A. Assi[1], Sylvia Hartmann[2], Arthur G. Riggs[3], Stephan Mathas[4,5], Peter N. Cockerill[1] and Constanze Bonifer[1]

[1]Institute for Cancer and Genomic Sciences, University of Birmingham, College of Medical and Dental Sciences, Birmingham B152TT, UK, [2]Senckenberg Institute of Pathology, University Hospital, Frankfurt, Germany, [3]Beckman Research Institute of City of Hope Medical Center, Duarte, CA 91010, USA, [4]Max-Delbrück-Center for Molecular Medicine, 13125 Berlin, Germany; [5]Hematology, Oncology, and Tumor Immunology, Charité – Universitätsmedizin Berlin, 12200 Berlin, Germany

Supplementary Materials

Table of Contents

1. Supplementary Figures 2
2. Supplementary Materials and Methods 17
3. Supplementary Tables 24
   Table 1. qPCR gene expression primers 24
   Table 2. siRNA 24
   Table 3. Western blotting antibodies. 25
   Table 4. Alignment rates of RACE-Seq experiments 25
   Table 5. Alignment rates of cell line RNA-Seq experiments 26
   Table 6. Alignment rates of LCM RNA-Seq experiments 26

1. Supplementary Figures

A THE1B Consensus Sequence

TGATATGGTTTGGCTGTGTCCCCACCCAAATCTCATCTTGAATTGTAGCTCCCATAATTCCCA
CGTGTCGTGGGAGGGACCCGGTGGGAGGTAATTGAATCATGGGGGCGGGTCTTTCCCGT
GCTGTTCTCGTGATAGTGAATAAGTCTCACGAGATCTGATGGTTTTATAAAGGGGAGTTYCC

Transcription Start Site

CTGCACAN***G***CTCTCTTGCCTGCCGCCATGTAAGACGTGMCTTTGCTCCTCCTTCGCCTTCY

THE1B Consensus Primer **Splice Site**

GCCATGATTG**TGAGGCCTCCCCAGCCATG**TGGAAC**TG**TGAGTCCATTAAACCTCTTTYCTTT
ATAAATTACCCAGTCTCGGGTATGTCTTTATTAGCAGCATGAGAACGGACTAATACA

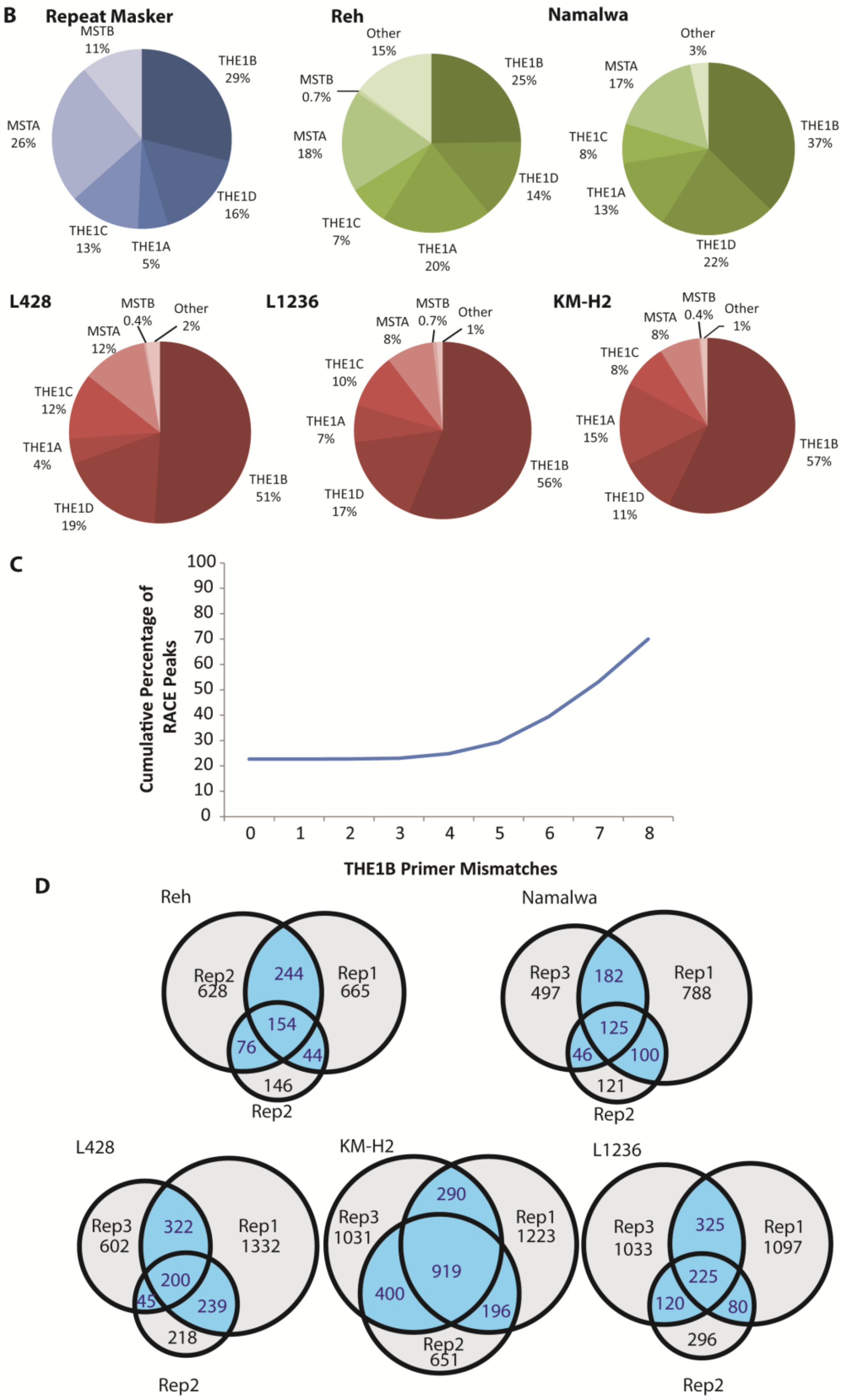

Figure S1. HL cell lines display a global activation of long terminal repeat elements A) Full THE1B consensus sequence showing predicted transcription start site, splice site and THE1B RACE primer location B) Annotation of RACE-Seq peaks with the Repeat Masker hg19 repeat family annotation. C) Comparison of the THE1B primer sequence to the genomic sequence of active LTRs identified by RACE-Seq. D) Overlap of peaks identified in biological RACE-Seq replicates. Highlighted areas, in blue, represent those peaks which are shared by at least 2 replicates in a cell line ($p<0.01$, hypergeometric analysis) and are the peaks which were selected for further downstream analysis.

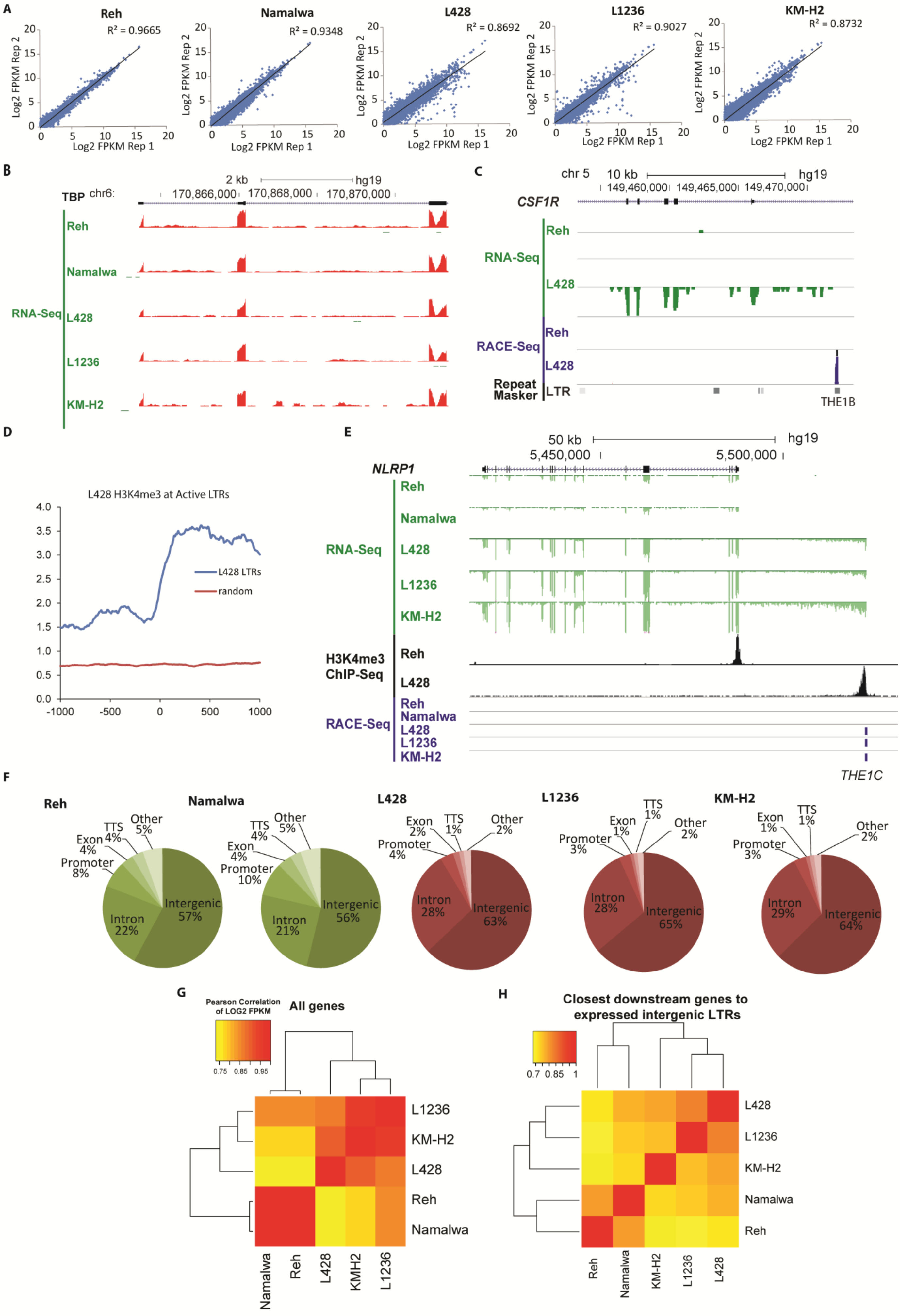
A
Reh
R² = 0.9665
Namalwa
R² = 0.9348
L428
R² = 0.8692
L1236
R² = 0.9027
KM-H2
R² = 0.8732
Log2 FPKM Rep 2
Log2 FPKM Rep 1
B
TBP chr6:
2 kb
hg19
170,866,000
170,868,000
170,870,000
RNA-Seq
Reh
Namalwa
L428
L1236
KM-H2
C
chr 5
10 kb
hg19
149,460,000
149,465,000
149,470,000
CSF1R
RNA-Seq
Reh
L428
RACE-Seq
Reh
L428
Repeat Masker
LTR
THE1B
D
L428 H3K4me3 at Active LTRs
L428 LTRs
random
E
50 kb
hg19
5,450,000
5,500,000
NLRP1
RNA-Seq
Reh
Namalwa
L428
L1236
KM-H2
H3K4me3 ChIP-Seq
Reh
L428
RACE-Seq
Reh
Namalwa
L428
L1236
KM-H2
THE1C
F
Reh
Other 5%
TTS 4%
Exon 4%
Promoter 8%
Intergenic 57%
Intron 22%
Namalwa
Other 5%
TTS 4%
Exon 4%
Promoter 10%
Intergenic 56%
Intron 21%
L428
Exon 2%
TTS 1%
Other 2%
Promoter 4%
Intron 28%
Intergenic 63%
L1236
TTS 1%
Exon 1%
Other 2%
Promoter 3%
Intron 28%
Intergenic 65%
KM-H2
Exon 1%
TTS 1%
Other 2%
Promoter 3%
Intron 29%
Intergenic 64%
G
Pearson Correlation of LOG2 FPKM
All genes
0.75 0.85 0.95
L1236
KM-H2
L428
Reh
Namalwa
KMH2
H
Closest downstream genes to expressed intergenic LTRs
0.7 0.85 1
L428
L1236
KM-H2
Namalwa
Reh

Figure S2. LTR activation contributes to global deregulation of gene expression in HL cell lines. A) Comparison of RNA-Seq biological replicates Log2 FPKM values for each gene with an FPKM of at least 1 were plotted and linear regression calculated. B) UCSC genome browser screenshot showing quality of RNA-Seq data. . C) UCSC genome browser screenshot showing a THE1B LTR acting as a promoter for the *CSF1R* gene in the L428 HL cell line. D) H3K4me3 ChIP-Seq signal in L428 cells centred on active LTRs identified by RACE-Seq and at control random sites E) UCSC genome browser screenshot showing H3K4me3 ChIP-Seq signal at the cHL specific THE1C LTR producing a transcript of *NLRP1* and at the endogenous promoter in Reh cells (1) F) Annotation of expressed LTRs identified by RACE-Seq to genomic regions in which they are located. G) Pearson correlation of gene expression patterns determined by RNA-Seq and clustered by hierarchical unsupervised clustering. H) The orientation of active LTRs identified by RACE-Seq was inferred from annotation of LTR orientation by repeat masker. The closest genes downstream of active LTRs were annotated and the expression values obtained from RNA-Seq data. The expression of these genes was correlated by Pearson correlation and the result clustered.

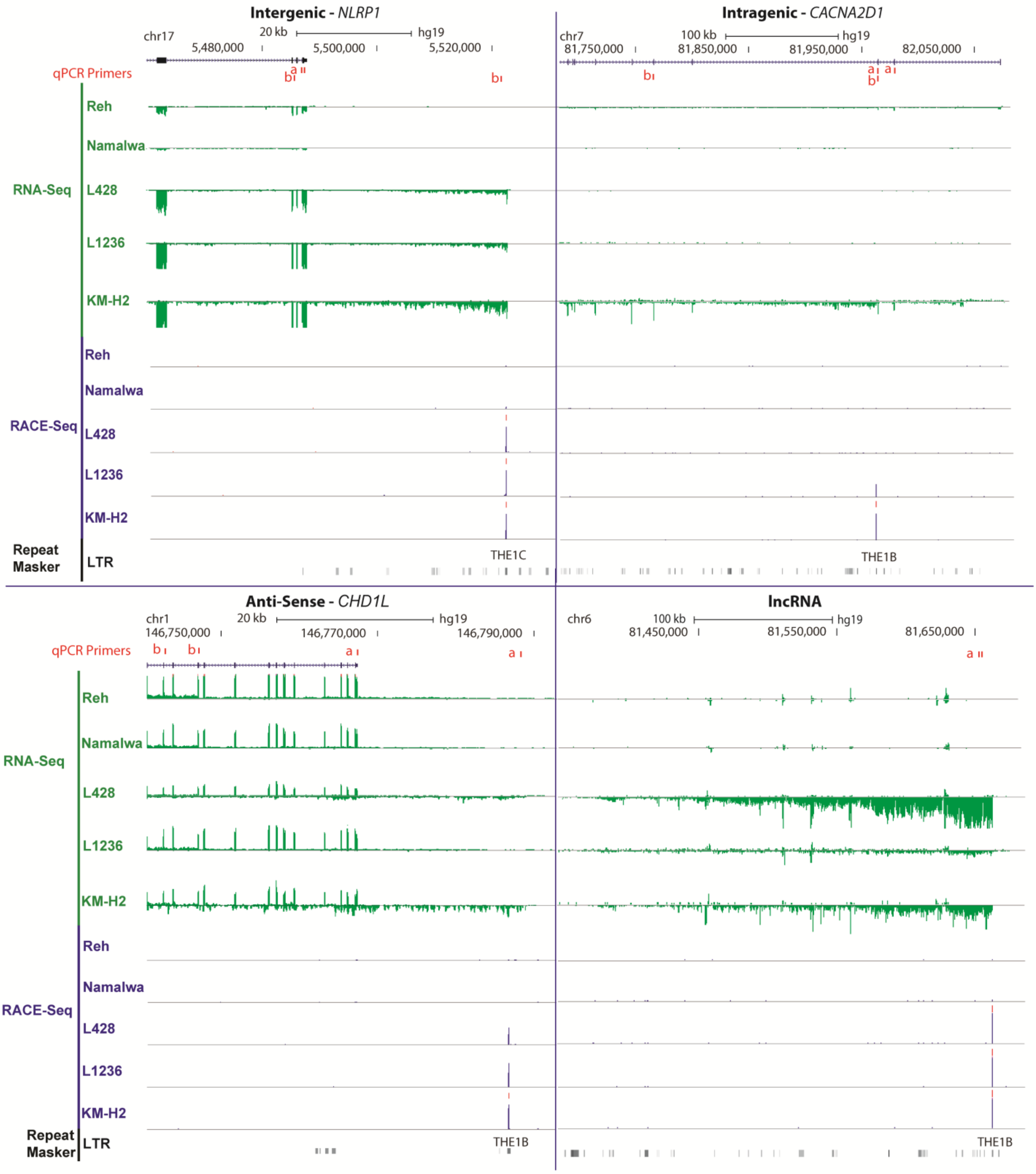

Figure S3. THE1 produced 4 types of transcript in HRS cell lines. UCSC genome browser screenshots showing aligned RNA-Seq and RACE-Seq reads showing examples of 4 types of transcript which originate from active THE1 LTRs in HRS.

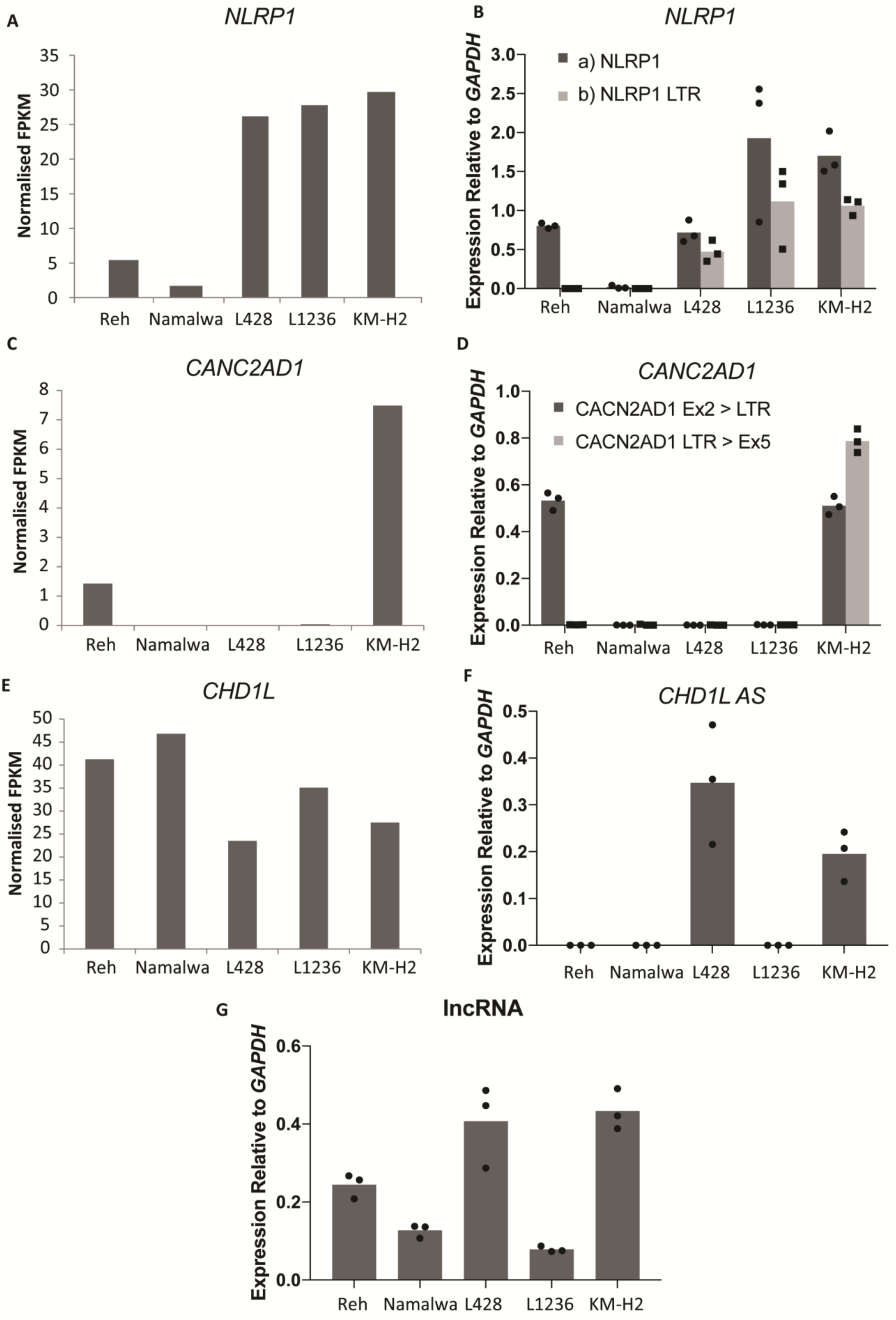
A
NLRP1
Normalised FPKM
35
30
25
20
15
10
5
0
Reh
Namalwa
L428
L1236
KM-H2
B
NLRP1
Expression Relative to GAPDH
3.0
2.5
2.0
1.5
1.0
0.5
0.0
a) NLRP1
b) NLRP1 LTR
Reh
Namalwa
L428
L1236
KM-H2
C
CANC2AD1
Normalised FPKM
8
7
6
5
4
3
2
1
0
Reh
Namalwa
L428
L1236
KM-H2
D
CANC2AD1
Expression Relative to GAPDH
1.0
0.8
0.6
0.4
0.2
0.0
CACN2AD1 Ex2 > LTR
CACN2AD1 LTR > Ex5
Reh
Namalwa
L428
L1236
KM-H2
E
CHD1L
Normalised FPKM
50
45
40
35
30
25
20
15
10
5
0
Reh
Namalwa
L428
L1236
KM-H2
F
CHD1L AS
Expression Relative to GAPDH
0.5
0.4
0.3
0.2
0.1
0.0
Reh
Namalwa
L428
L1236
KM-H2
G
lncRNA
Expression Relative to GAPDH
0.6
0.4
0.2
0.0
Reh
Namalwa
L428
L1236
KM-H2

Figure S4. THE1 produced 4 types of transcript in HL cell lines. A) Normalised RNA-Seq FPKM values showing expression of *NLRP1*. B) qPCR gene expression analysis using primers designed in exon 1 ($p<.05$ L1236 and KM-H2 vs Reh and Namalwa) and between the upstream LTR and Exon 2 ($p<.05$ HL cell lines vs control cell lines, paired Student t test). C) Normalised RNA-Seq FPKM values showing expression of CACN2AD1. D) qPCR gene expression analysis using primers designed for transcripts before and after the intragenic LTR ($p<.01$ KM-H2 compared to all other cell lines, paired Student t test). E) Normalised RNA-Seq FPKM values showing expression of *CHD1L.* F) qPCR gene expression analysis using primers designed in the LTR driven anti-sense transcript ($p<.01$, L428 and KM-H2 vs L1236 and control, paired Student t test) . G) qPCR gene expression analysis using primers designed in an LTR driven lncRNA transcript ($p<.01$, HL vs control cell lines, paired Students t test).

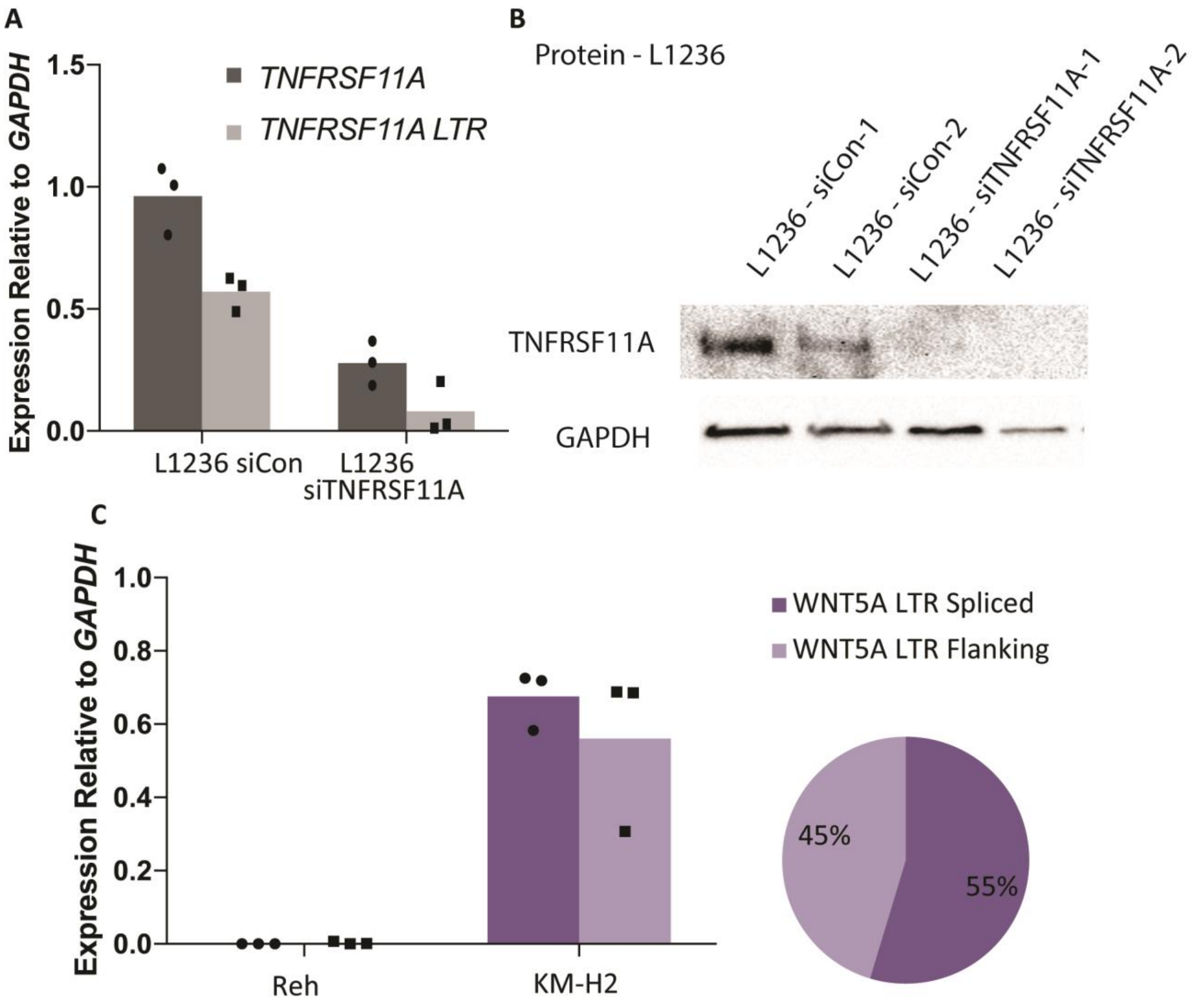

Figure S5. TNFRSF11A-LTR transcript is downregulated by siRNA targeting exon 7 of TNFRSF11A and the WNT5A-LTR has a spliced and lncRNA transcript. A) qPCR gene expression analysis showing expression of a transcript between exon 2 & 3 and also between the upstream LTR and exon 2 following siRNA knockdown compared to non-targeting control. Error bars show standard deviation from 3 biological replicates (p<.05 L1236 vs control cell lines, paired Student t test). B) TNFRSF11A protein measured by Western blot following siRNA knockdown compared to non-targeting control. C) Spliced and un-spliced WNT5A-LTR transcript quantified by qPCR. Error bars show standard deviation of 3 biological replicates.

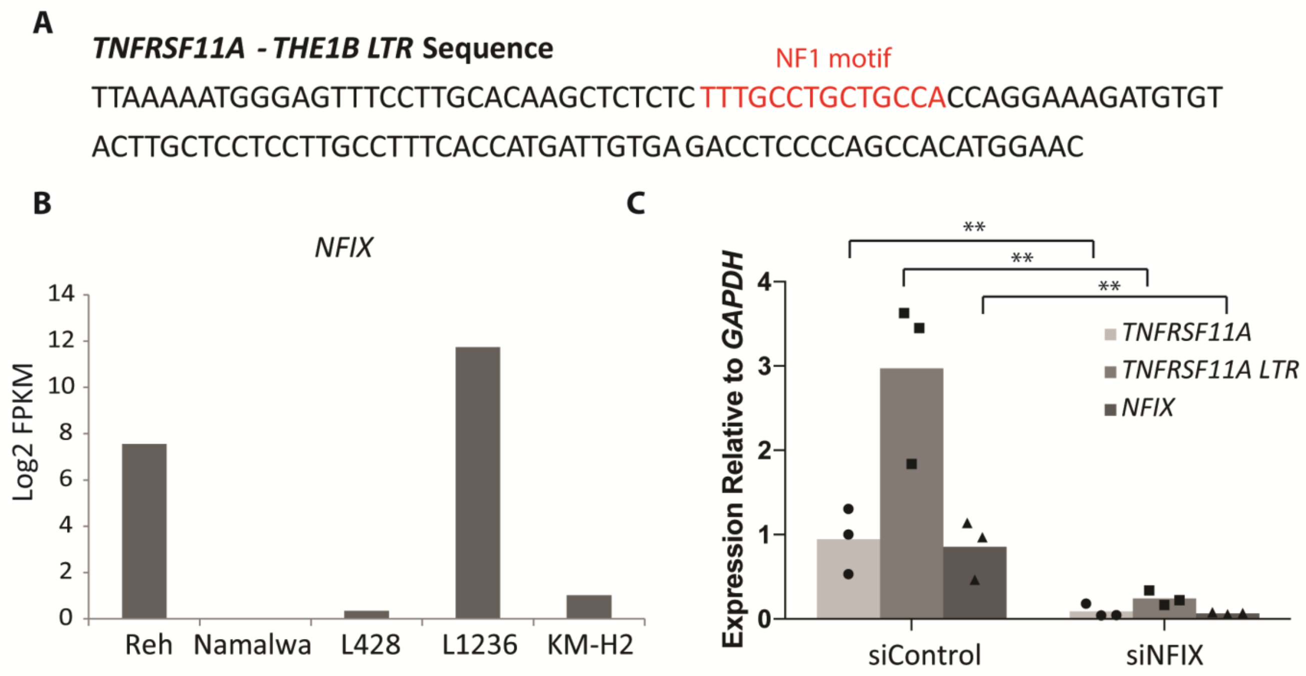

Figure S6. NFIX expression is required for *TNFRSF11A-LTR* activation. A) *TNRFRSF11A-LTR* sequence showing presence of NF1 motif. B) Normalised RNA-Seq FPKM values showing expression of *NFIX*. C) qPCR gene expression analysis showing expression of *TNFRSF11A*, *TNFRSF11A-LTR* and *NFIX* transcripts following siRNA knockdown of NFIX compared to non-targeting control. Error bars show standard deviation from 3 biological replicates ($p<.05$ L1236 vs control cell lines, paired Student t test).

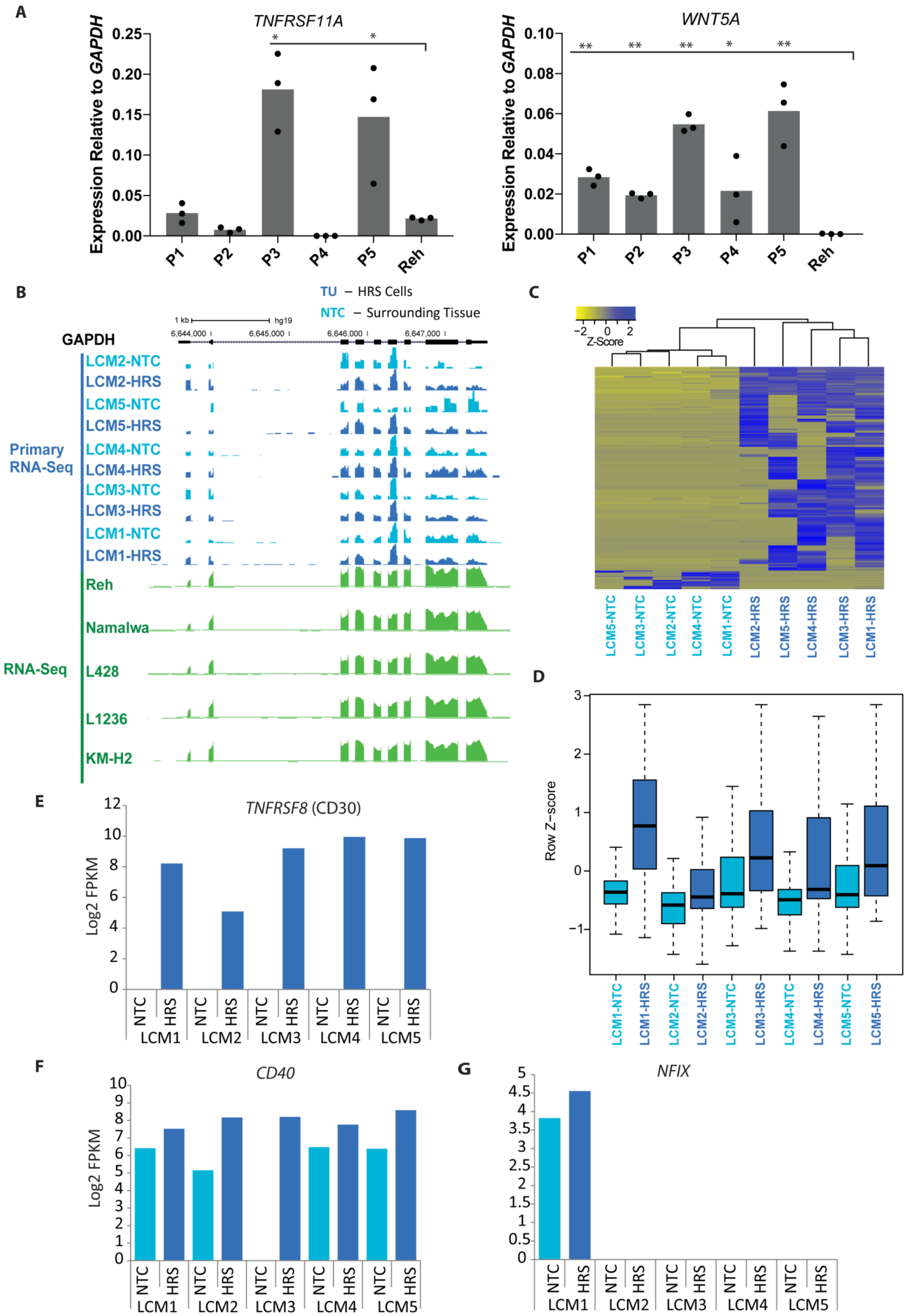
A
TNFRSF11A
Expression Relative to GAPDH
P1
P2
P3
P4
P5
Reh
WNT5A
B
TU – HRS Cells
NTC – Surrounding Tissue
GAPDH
LCM2-NTC
LCM2-HRS
LCM5-NTC
LCM5-HRS
LCM4-NTC
LCM4-HRS
LCM3-NTC
LCM3-HRS
LCM1-NTC
LCM1-HRS
Primary RNA-Seq
Reh
Namalwa
L428
L1236
KM-H2
RNA-Seq
C
Z-Score
D
Row Z-score
E
TNFRSF8 (CD30)
Log2 FPKM
NTC
HRS
LCM1
LCM2
LCM3
LCM4
LCM5
F
CD40
G
NFIX

Figure S7. *TNFRSF11A* and *WNT5A LTR* transcripts can be detected in cHL tumour tissue and HRS cells. A) qPCR measuring *TNFRSF11A* expression performed on RNA extracted from frozen HRS tumour samples (P1 – P5) and Reh (negative control). Error bars show standard deviation from n=3. (*p<.05 paired Student t test). B) UCSC genome browser screenshot showing alignment of RNA-Seq from laser micro-dissected HRS cells and bystander cells (NTC) from cHL tumour samples. Cell line RNA-Seq is shown for comparison at the *GAPDH* gene. C) Clustering of row z-scores of common HRS versus NTC differentially regulated genes in HRS and NTC samples D) Boxplots showing a comparison the row z-scores of the 150 most up-regulated genes from (2) to LCM RNA-Seq data in HRS and bystander cells (NTC). E) Normalised RNA-Seq FPKM values showing expression of *TNFRSF8*. F) Normalised RNA-Seq FPKM values showing expression of *CD40.* G) Normalised RNA-Seq FPKM values showing expression of *NFIX*.

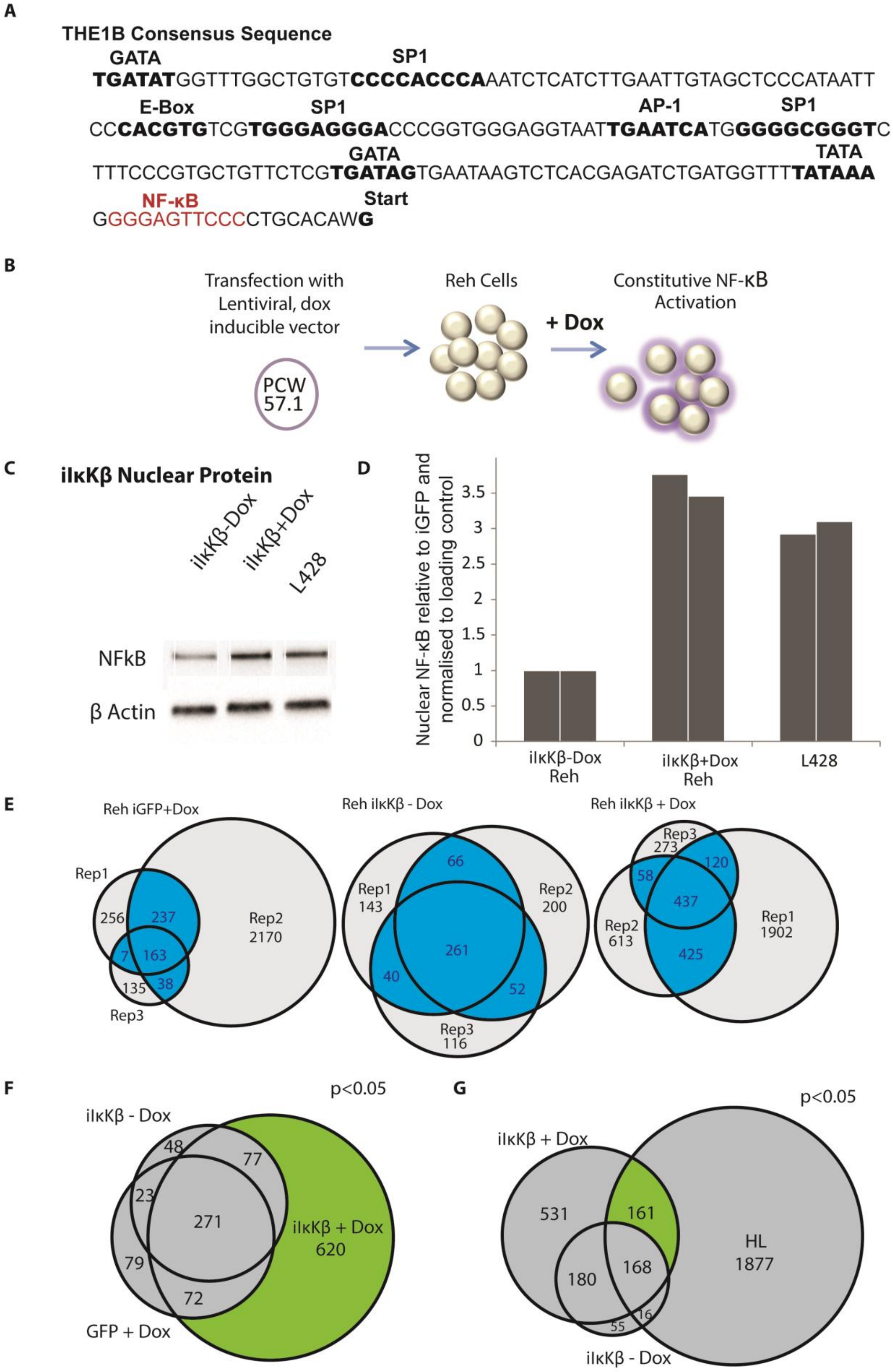
A
THE1B Consensus Sequence
GATA
SP1
TGATATGGTTTGGCTGTGTCCCCACCCAAATCTCATCTTGAATTGTAGCTCCCATAATT
E-Box
SP1
AP-1
SP1
CCCACGTGTCGTGGGAGGGACCCGGTGGGAGGTAATTGAATCATGGGGGCGGGTC
GATA
TATA
TTTCCCGTGCTGTTCTCGTGATAGTGAATAAGTCTCACGAGATCTGATGGTTTTATAAA
NF-κB
Start
GGGGAGTTCCCCTGCACAWG
B
Transfection with Lentiviral, dox inducible vector
PCW 57.1
Reh Cells
+ Dox
Constitutive NF-κB Activation
C
iIκKβ Nuclear Protein
iIκKβ-Dox
iIκKβ+Dox
L428
NFkB
β Actin
D
Nuclear NF-κB relative to iGFP and normalised to loading control
0
0.5
1
1.5
2
2.5
3
3.5
iIκKβ-Dox Reh
iIκKβ+Dox Reh
L428
E
Reh iGFP+Dox
Rep1
256
237
7
163
38
135
Rep3
Rep2 2170
Reh iIκKβ - Dox
66
Rep1 143
Rep2 200
261
40
52
Rep3 116
Reh iIκKβ + Dox
Rep3 273
58
120
437
Rep2 613
425
Rep1 1902
F
p<0.05
iIκKβ - Dox
48
77
23
271
79
72
iIκKβ + Dox 620
GFP + Dox
G
p<0.05
iIκKβ + Dox
531
161
180
168
HL 1877
55
16
iIκKβ - Dox

Figure S8. Constitutive NF-κB activation drives expression of a set of THE1 LTRs. A) THE1B LTR consensus sequence obtained from RepBase and annotated with transcription factor binding motifs, highlighting the presence of an NF-κB motif. B) Doxycycline (Dox) inducible NF-κB activation scheme. C) NF-κB activation in Reh cells confirmed by nuclear localisation of NF-κB as measured by western blotting. D) Relative quantification of inducible nuclear NF-κB localisation in Reh cells as measured by densitometry of western blots. E) Overlap of active LTR peaks identified by THE1B RACE-Seq in 3 biological replicates. F) Overlap of RACE-Seq LTR peaks before and after NF-κB activation. G) Overlap of RACE-Seq LTR peaks after NF-κB activation with merged peaks from the 3 HRS cell lines.

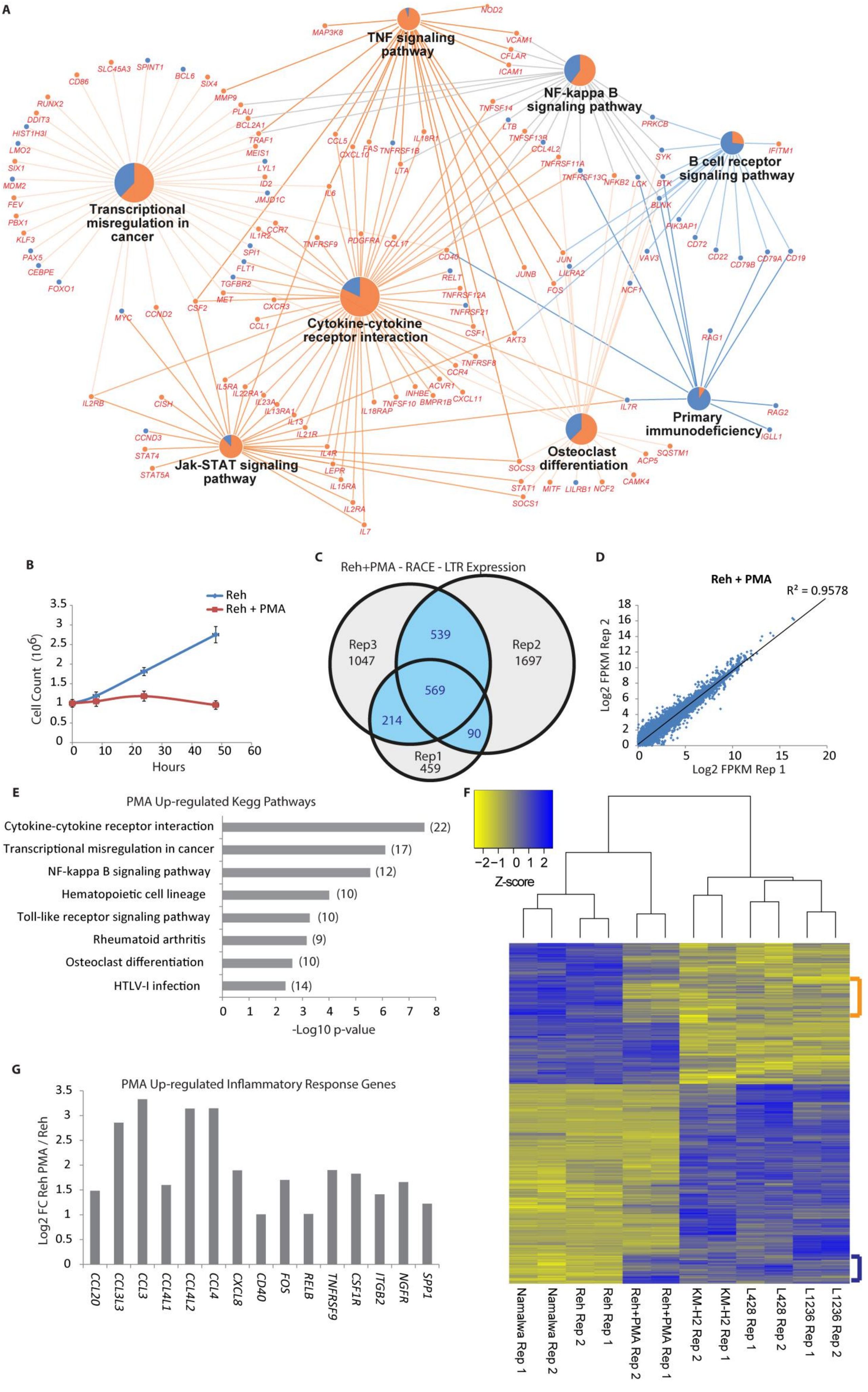
A
TNF signaling pathway
NF-kappa B signaling pathway
B cell receptor signaling pathway
Transcriptional misregulation in cancer
Cytokine-cytokine receptor interaction
Jak-STAT signaling pathway
Osteoclast differentiation
Primary immunodeficiency
NOD2
MAP3K8
VCAM1
CFLAR
ICAM1
SLC45A3
SPINT1
BCL6
SIX4
CD86
MMP9
RUNX2
PLAU
DDIT3
BCL2A1
HIST1H3I
TRAF1
LMO2
MEIS1
SIX1
LYL1
MDM2
ID2
FEV
JMJD1C
PBX1
KLF3
PAX5
CEBPE
FOXO1
TNFSF14
LTB
TNFSF13B
CCL4L2
TNFRSF11A
TNFRSF13C
NFKB2
LCK
BTK
PRKCB
SYK
IFITM1
BLNK
PIK3AP1
CD72
CD22
CD79B
CD79A
CD19
VAV3
NCF1
RAG1
RAG2
IGLL1
CCL5
CXCL10
FAS
TNFRSF1B
IL18R1
LTA
IL6
CCR7
IL1R2
SPI1
FLT1
TGFBR2
MET
CXCR3
CCL1
TNFRSF9
PDGFRA
CCL17
CD40
RELT
TNFRSF12A
TNFRSF21
CSF1
AKT3
JUNB
JUN
LILRA2
FOS
MYC
CCND2
CSF2
IL2RB
CISH
CCND3
STAT4
STAT5A
IL5RA
IL22RA1
IL23A
IL13RA1
IL13
IL21R
IL4R
LEPR
IL15RA
IL2RA
IL7
IL18RAP
TNFSF10
INHBE
BMPR1B
ACVR1
CXCL11
CCR4
TNFRSF8
IL7R
SOCS3
STAT1
SOCS1
MITF
LILRB1
NCF2
CAMK4
ACP5
SQSTM1
B
Reh
Reh + PMA
Cell Count (10^6)
Hours
0 10 20 30 40 50 60
0 0.5 1 1.5 2 2.5 3 3.5
C
Reh+PMA - RACE - LTR Expression
Rep3 1047
Rep2 1697
Rep1 459
539
569
214
90
D
Reh + PMA
R² = 0.9578
Log2 FPKM Rep 2
Log2 FPKM Rep 1
0 5 10 15 20
0 2 4 6 8 10 12 14 16 18
E
PMA Up-regulated Kegg Pathways
Cytokine-cytokine receptor interaction (22)
Transcriptional misregulation in cancer (17)
NF-kappa B signaling pathway (12)
Hematopoietic cell lineage (10)
Toll-like receptor signaling pathway (10)
Rheumatoid arthritis (9)
Osteoclast differentiation (10)
HTLV-I infection (14)
0 1 2 3 4 5 6 7 8
-Log10 p-value
F
−2 −1 0 1 2
Z-score
Namalwa Rep 1
Namalwa Rep 2
Reh Rep 2
Reh Rep 1
Reh+PMA Rep 2
Reh+PMA Rep 1
KM-H2 Rep 2
KM-H2 Rep 1
L428 Rep 1
L428 Rep 2
L1236 Rep 1
L1236 Rep 2
G
PMA Up-regulated Inflammatory Response Genes
Log2 FC Reh PMA / Reh
0 0.5 1 1.5 2 2.5 3 3.5
CCL20
CCL3L3
CCL3
CCL4L1
CCL4L2
CCL4
CXCL8
CD40
FOS
RELB
TNFRSF9
CSF1R
ITGB2
NGFR
SPP1

Figure S9. Treatment of the Reh cell line with PMA induces global THE1B LTR activation. A) KEGG pathway analysis linking 2-fold dysregulated genes in HL was plotted as a network to show genes which are shared between multiple pathways. Orange represents genes which are up-regulated in at least one HL cell line compared to the control cell lines (Reh and Namalwa) and blue represents genes which are down-regulated. The pie charts and colour of the lines for each pathway indicate the proportion of the dysregulated genes which are up- (orange) or down-regulated (blue) in that pathway. B) Growth curve following treatment of Reh cells with 2ng/ml. Error bars show standard deviation over 3 biological replicates. C) Overlap of active LTR peaks identified by THE1B RACE-Seq following treatment of Reh cells following treatment of Reh cells with 2ng/ml for 8 hours in 3 biological replicates. D) Comparison of RNA-Seq biological replicates Log2 FPKM values for each gene with an FPKM of at least 1 were plotted and linear regression calculated. E) KEGG pathway analysis for genes up-regulated following treatment of Reh cells F) Differential gene expression measured by RNA-Seq represented by row z-score. Bracket show differentially expressed genes shared between Reh cells following PMA treatment and cHL cell lines (orange – downregulated, blue – upregulated). G) Example up-regulated inflammatory response genes following PMA treatment of Reh cells.

2. Supplementary Materials and Methods

5' RACE

RACE was performed using the THE1B specific primer CATGGCTGGGGAGGCCTCA and 5' RACE adaptor TCATACACATACGATTTAGGTGACACTATAGAGCGGCCGCCTGCAGGAAA to amplify cDNA products containing a conserved region of the THE1B LTR and the transcription start site. RACE was carried out based on the ExactSTART™ Eukaryotic mRNA 5'-& 3'-RACE Kit (Epicentre) using the supplied protocol. However, due to the discontinuation of supply of the Tobacco Acid Pyrophosphatase enzyme, a number of modifications were made.

To perform 5' RACE RNA was treated with Alkaline Phosphatase for 15 minutes at 37°C (10 µl Apex Buffer, 5 µl Apex Heat-liable Alkaline Phosphatase (Epicentre), 1 µg RNA, made up to 100 µl with H_2O). An Ampure RNA-clean bead purification was then carried out prior to treatment with RNA 5' Pyrophosphohydrolase (RppH) for 1 hour at 37 °C (Purified RNA (40 µl), 10 x Thermopol Buffer (NEB)(5 µl), RppH Enzyme (NEB)(5 µl)). The RppH reaction was stopped by addition of 1 µl of 500 mM EDTA solution and then purified with a further Ampure RNA-clean bead purification and eluted in 16 µl H_20. The 5' RACE acceptor oligonucleotide was ligated at 37 °C for 30 minutes after addition of 4 µl H_2O, 2 µl RNA ligase buffer, 1 µl 5' acceptor oligonucleotide, 1 µl 2mM ATP solution and 1 µl T4 RNA ligase (NEB). First strand cDNA synthesis was carried out by addition of 14 µl H_2O, 1 µl cDNA synthesis primer, 2 µl dNTP PreMix, 2 µl MMLV RT Buffer and 1 µl MMLV RT, the reaction was incubated at 37°C for 1 hour followed by 85°C for 10 minutes. The reaction then had 1 µl of RNase added and was incubated at 55°C for 5 minutes. The second strand cDNA synthesis reaction components were added at 55°C. The consensus biotinylated THE1B LTR primer CATGGCTGGGGAGGCCTCA (20 µM, 2 µl) was used along with a primer complementary to the previously ligated 5' RACE adaptor (5 µl), 21 µl H_2O, 30 µl PCR mix and 1 µl (2.5 U) PfuUltra II polymerase and incubated for 21 cycles (95°C 30 seconds, 21 x 95°C 20 seconds, 60 °C 20 seconds, 72°C 3 minutes).

Following second strand synthesis the fragments incorporating a biotinylated primer were selected using T1 Dynabeads (Thermo Fisher Scientific) as follows. T1 dynabeads (20 µl) were washed in B&W buffer (10 mM Tris-HCL, 1 mM EDTA, 2 M NaCl) using magnetic separation and re-suspended in 50 µl of 2 x buffer B&W buffer. An equal volume of RACE product was added and samples were mixed on a slow rotating wheel at room temperature for 1 hour. The beads were then captured using a magnetic separator washed with B&W buffer and TE and were re-suspended in 33.75 µl 1 x TE.

The selected DNA was amplified off the beads by a 3 cycle PCR (95°C 2 minutes, 3 x 95°C 20 seconds, 56 °C 20 seconds, 72°C 30 seconds and 72°C 3 minutes) using a non-biotinylated THE1B LTR primer (1.25 µl, 20µM), the 5' RACE adaptor primer (4 µl), 5 µl dNTPs (10mM), 5 µl PFU Ultra Buffer and 1 µl PFU Ultra polymerase. Finally purification was carried out using the Qiagen MiniElute PCR Cleanup Kit and the final product was eluted in 11 µl elution buffer.

RACE-Seq

Libraries for genome wide RACE-Seq were produced using the MicroPlex Library Preparation Kit v2 (Diagenode). Purified RACE material (10 µl) was added to 2 µl of template preparation buffer and 1 µl of Template Preparation Enzyme and incubated at 22°C for 25 minutes followed by 55°C for 20 minutes. The prepared template was then mixed with 1 µl Library synthesis buffer according

to the manufacturer's instructions and 1 µl Library synthesis enzyme and incubated at 22°C for 40 minutes. The libraries were then mixed with amplification buffer (25 µl), amplification enzyme (1 µl), H_2O (4 µl) and a Barcoded Indexing Reagent (5 µl) to allow for the samples to be multiplexed for sequencing. The mix was split into 2 reactions of 25 µl which were amplified at 12 and 14 cycles to allow for selection of enough material to sequence without introducing clonal amplification (Extension and Cleavage: 72°C 3 minutes, 85°C 2 minutes, Denaturation: 98°C 2 minutes, Addition of Indexed oligonucleotides: 4 x 98°C 20 seconds, 67°C 20 seconds, 72°C 40 seconds, Library Amplification: 12 or 14 x 98°C 20 seconds, 72°C 50 seconds).

Finally size selection and purification of the libraries was carried out by running products on a 1.2% TAE agarose gel (with 0.05% ethidium bromide) and excising fragments between 190 and 300 bp. These were then extracted using the Qiagen mini-elute gel extraction kit and eluted twice in 12 µl H_2O. The libraries were run on a Bioanalyzer 2100 with a High Sensitivity DNA Assay chip (Agilent) to determine the average fragment size and quantified by PCR using the Kappa Illumina Library Quantification Kit on an Applied Biosystems StepOne Plus RT PCR system.

The indexed libraries were pooled and sequenced on Illumina MiSeq using the 150-Cycle paired end kit or a NextSeq 500 as a fraction of a 150 cycle flow cell.

ChIP-Seq

For ChIP, $5x10^6$ L428 cells were washed, resuspended at $3.3x10^6$ cells/ml and initially cross-linked with 8.3 µl/ml DSG for 45 mins at room temperature. Cells were then washed 3 times with PBS using 500 g centrifugation, then subsequently crosslinked in 1% formaldehyde for 10 mins at room temperature. Quenching was carried out in by adding 1/10 volume of 2M glycine for a final concentration of 0.2M. Cells were Washed twice with ice-cold PBS, resuspended in1 ml 10 mM Hepes, 10 mM EDTA, 0.5 mM EGTA (all ph 8.0), 0.25 % Triton X100 and rotated 4 °C for 10 mins for lysis. Cells were then centrifuged at 500 g at 4 °C for 5 mins and nuclei resuspended in in 1ml 10 mM Hepes, 1mM EDTA, 0.5 mM EGTA (all pH 8.0), 200 mM NaCl, 0.01 % Triton X100 and rotated 4°C for 10 mins. Nuclei were then centrifuged at 500 g at 4°C for 5 mins and resuspended in 150 µl 25 mM Tris, 2 mM (both pH 8.0), 150 mM NaCl, 1 % Triton X100, 0.25 % SDS Sonication was performed using a Picoruptor sonicator (Diagenode) using 30 30s on, 30s off cycles. Sonicated chromatin was centrifugated for 10 min at 16,000 g at 4 °C to pellet debris, then diluted in 300 µl 25 mM Tris, 2 mM (both pH 8.0), 150 mM NaCl, 1 % Triton X100, 0.25 %, 7.5 % Gylcerol (final volume 450 µl ,0.083 % SDS, 5 % glycerol final concentration). All buffers contained 1:100 phosphatase inhibitor cocktail (Sigma-Aldrich) and 0.1 mM PMSF. 5% of the sonicated chromatin was saved as input. 15 µl Protein G Dynabeads (Thermo Fisher Scientific) were washed with 500 µl PBS + 0.0 2% Tween 20, resuspended in 15 µl PBS + 0.02 % Tween 20, 0.5 % BSA and 2 µg H3K4me3 millipore 04745 antibody, and rotated at 4 °C for 2 hours. Beads were washed with 500 µl PBS + 0.02 % Tween 20 and incubated with the sonicated chromatin (400 µl) on a rotating wheel overnight at 4 °C. Beads were then washed twice with 1ml 20 mM Tris, 2 mM EDTA 0.5 M (both pH 8.0), 150 mM NaCl, 1 % TritonX100, 0.1 % SDS, once with 1ml 20 mM Tris, 2 mM EDTA 0.5 M (both pH 8.0), 500 mM NaCl, 1 % TritonX100, 0.1 % SDS, once with 1 ml 10 mM Tris, 1 mM EDTA (both pH 8.0), 250 mM LiCl 0.5 % NP40, 0.5 % Na-deoxycholate and twice with 1ml 10 mM Tris, 1 mM EDTA (both pH 8.0), 50 mM NaCl. Beads were subsequently incubated twice with 50 µl 100 mM $NaHCO_3$, 1% SDS at 65 °C, and eluates reverse crosslinked overnight with 200 mM NaCl, 0.25 µg/µl proteinase K at 65 °C. Eluates were then incubated 1 hr with 0.1 µg/µl RNAse A at 37 °C. Phenol-chloroform extraction was performed by adding twice 100 µl equilibrated phenol-

chloroform isoamyl alcohol (25:24:1), vortexing for 30s and spinning down at 16,000 g at 4°C for 5 mins, then adding 2.5 volumes 100% ethanol, 1/10 volume 5M NaCl and 1 µl glycogen, resuspending in 100 µl 0.1 H_2O.

Libraries were generated using the Kapa Hyperkit protocol (Kapa Biosystems) according to manufacturer's instructions, using 16 cycles of amplification. 200-400 bp fragments were size-selected using a 2% agarose gel then subsequently purified using the QiaQuick Gel Extraction kit (QiaGen) according to manufacturer's instructions. High-throughput sequencing was performed on an Illumina HiSeq 2500 sequencer (Illumina, USA).

Data Analysis

RNA-Seq

For RNA-Seq performed in cell lines, reads were mapped to the hg19 human reference genome using Tophat2 (3) using --library-type fr-firstrand (for alignment rates see Supp. Table 5). Reads mapping to the sense and anti-sense strands were split into separate files using the following commands: samtools view -b -f 128 -F 16 and samtools view -b -f 80 for forward reads; samtools view -b -f 144 and samtools view -b -f 64 -F 16 for reverse reads (4). Bam files generated by separate samtools commands were subsequently merged by forward or reverse read status using samtoos merge. and histogram density plots were created from the mapped reads using bedtools genomecov with the '-d -split' option and uploaded to UCSC genome browser (5). To obtain normalised FPKM (fragments per kilobase of transcript per million mapped reads) values for gene expression using cuffnorm (6) with --library-type fr-firststrand. Further analysis was carried out using $\log_2$ FPKM values in R and Microsoft Excel (7). Expressed genes were defined as any gene with a $\log_2$ FPKM value of 0 or above and differential expression between cell lines was defined based on at least a 2-fold change in expression.

For RNA-Seq performed in laser capture micro-dissected primary material, reads were mapped to the hg19 human reference genome using hisat2 (8) using default parameters (for alignment rates see Supp. Table 6). To account for noise due to low cell numbers and to remove artefact reads in exons, FPKM values were computed using DESeq2 (9) following read count per feature using featureCounts --countSplitAlignmentsOnly to exclude artefacts corresponding to PCR duplicates in exons originating from genomic DNA (10).

To perform clustering of the RNA-seq data from the cell lines pairwise Pearson correlation of gene expression was used to produce a correlation matrix. Clustering of the RNA-Seq data by Pearson correlation was performed using R with the heatmap.2 function in the gplots package using hierarchical clustering with Euclidean distance and average linkage. For correlations of replicates, spearman correlation coefficients of $\log_2$(FPKM+1) values were used then clustered using the heatmap.2 function of the gplots package.

For DRG heatmaps from cell line RNA-Seq, DRGs were computed with cuffdiff for all pairwise HL vs NHL comparisons, i.e. all pairwise combinations of KM-H2, L1236, L428 versus Namalwa, Reh, using --library-type fr-firstrand as a parameter. Common HL upregulated genes were defined as significantly differentially regulated in all comparisons, i.e. $q<0.05$, intersecting using all up- and down- regulated genes from each comparison using the merge function of R, resulting in two lists

corresponding to common up- and down- regulated genes. For DRG heatmaps for patient RNA-Seq, TU vs NTC DRGs were computed via DESeq2, using a p-adjusted cutoff of 0.5. Row Z-scores were computed from FPKM values derived from cuffnorm as described above, using the following code in R: Z=t(scale(t(log(FPKM+1,2)),scale=T,center=T)). Heatmaps were plotted using the heatmap.2 function of the gplots package. For boxplots comparing the expression profiles from previously published LCM primary material microarray (2) and our own LCM primary material RNA-Seq, row Z-scores were described above retrieved for the top 150 upregulated genes from (2) and plotted for each sample using the boxplot function in R.

Gene Ontology analyses

Gene Ontology analysis was performed using DAVID on lists of up and down regulated genes as previously defined (11, 12). KEGG Pathway analysis was carried out using the ClueGo and CluePedia packages in Cytoscape with lists of up and down regulated genes combined from each HL cell line compared to each control cell line (13-15). The network was produced based on KEGG terms with a pV < 0.05 and the layout was manually adjusted to enable all interactions to be visualised.

RACE-Seq

RACE-Seq reads were first trimmed using nested cutadapt -g <adaptor> commands to remove the RACE adaptor sequence (TCATACACATACGATTTAGGTGACACTATAGAGCGGCCGCCTGCAGGAAA) and the THE1B consensus sequence (TGAGGCCTCCCCAGCCATG). The trimmed reads were then mapped to the hg19 version of the human reference genome using Bowtie2 in paired end mode with the '--very-sensitive' parameter (16) (for alignment rates see Supp. Table 4). Multi-mapping reads were removed using samtools view -bq 2. Histogram density plots were produced for each biological replicate and for the merged replicates using bedtools genomecov and the resulting plots uploaded to UCSC genome browser. Regions of enrichment (peaks) were identified using Macs1.4 with the '--keep-dup=all' parameter. The resulting peaks from each biological replicate were overlapped to identify the shared peaks using the ChipPeakAnno package in R and venn diagrams produced. High-confidence RACE-Seq peaks were defined by the presence of a peak in at least 2 out of 3 biological replicates. High confidence peaks were selected using an in house bedtools script (utilising nested bedtools intersect commands) and used for all further analysis. All further venn diagrams comparing the RACE-Seq peaks between cell lines were performed using the ChipPeakAnno package in R and lists of overlapping and specific peaks were created using the intersect function in bedtools. Annotation of repeat elements also made use of the bedtools intersect function overlapping the datasets with the Repeat Masker annotation track obtained from UCSC genome browser.

The clustering of RACE data between cell lines was carried out by creating a matrix of the number of peaks shared between each pair of cell lines using the ChipPeakAnno package in R (17). To compare these binary datasets the Dice index coefficient was calculated for each pairwise comparison in the context of the entire population and clustering was carried out as previously described using the heatmap.2 function in the gplots package of R.

Annotation of the genomic regions in which the active LTRs (RACE peaks) resided was performed using the annotatePeaks function of Homer. Annotation of LTR peaks to repeat elements was carried out using the command bedtools closest -a KM-H2_peaks_in_min_2reps_peaks.bed -b

../../Annotations/hg19_rmsk.bed -t first -D b | awk ' $10==0 '. LTR element types were counted by aggregating the resulting object in R using the aggregate function.

Closest genes to RACE peaks were identified using bedtools closest and a hg19 gene annotation reference. To determine closest genes in the same orientation and on the same strand the expressed LTR strand was first determined using bedtools intersect with the '-wao' parameter to obtain strand annotation form the repeat masker annotation. LTRs whose closest downstream gene shared the same strand, excluding LTRs located within genes, promoter and 3' UTR regions were selected and annotated usingbedtools slop -i hg19_refFlat.bed –b 1000 –g hg19.chrom.sizes | bedtools intersect -a <LTR peaks> -b - -v | bedtools closest -t first -iu -D a | awk ' $6==$12 '. The union of all closest genes was computed using the following command: cat <all LTR peaks with closest gene on same strand bed files> | awk '{ print $10 }' | sort | uniq. Files were subsequently split by strand using grep + or grep - commands. Average profiles were obtained using annotatePeaks < LTR peaks with closest gene on same strand split by + or – strand> hg19 -hist 10 -bedGraph <plus strand bedGraph> <,inus strand bedGraph> -size 500, by combining profiles with of matching and opposite strands, respectively, i.e. average profiles on the + and – strands for LTRs on the + and – strands, respectively, and on the + and – strands for LTRs on the – and + strands, respectively.

ChIP-Seq

Alignment was performed using Bowtie2 with -x hg19 --very-sensitive-local as parameters . Peak detection and coverage track generation was carried out using MACS with -t <bam> -n <name> -g hs --keep-dup=auto -w –S as parameters. For average H3K4me3 profiles, average read counts were obtained using annotatePeaks with -hist 10 -size 2000 as parameters, around L428 LTRs mapped to strand by annotating to repeat elements as described above, and around 10,000 random elements as a control, generated using bedtools random –g hg19.chrom.sizes -l 100 -n 10000.

LTR presence by gene expression fold change

Pairwise RNA-Seq datasets were ranked by log$_2$ FPKM fold change, defined as FC=(log$_2$ sample A FPKM+1)/(log$_2$ sample B FPMK+1) to avoid dividing by 0, with all genes ranked accordingly. Separately, LTRs identified via RACE-Seq were annotated to the closest gene using bedtools closest -a <LTR peak file.bed> -b hg19_refGene.bed -t first as parameters (5). LTR presence for all genes was thus computed by performing a left outer join of all genes and genes annotated as closest to LTR peaks via the merge function of R, using merge(<all genes sorted by log$_2$ FPKM fold change>, <gene list of annotated LTR peak file>, all.x=T), then replacing all matches with the value 1 and NULL values by 0 in the column originating from the gene list of the annotated LTR peak file. Resulting files were subsequently written as text files via write.table in R, then visualised and saved as heatmap images using Java TreeView (18). To test for significance of enrichment between the presence of LTRs and up- and down-regulated genes, hypergeometric tests were carried out in R as follows: p=1-phyper(<overlap>,<LTR>,<total>-<LTR>,<DEG>) where <overlap> is the overlap of gene names of 1 log$_2$ FPKM fold change up- or down-regulated genes, <LTR> the total number of genes closest to LTRs, <total> the number of genes in the hg19_refFlat annotation, and <DEG> the number of 1 log$_2$ FPKM fold change up- or down-regulated genes.

Public dataset processing

Microarray data from laser capture micro-dissected was obtained from GEO (GSE39133) (2). The data were normalised using ArrayAnalysis.org (19) and the top 150 up-regulated genes were chosen for comparison to the upregulated gene in our LCM RNA-Seq data. H3K4me3 ChIP-Seq performed in Reh cells was retrieved from GEO (GSE67540)(1) and processed as the ChIP-Seq data in this study.

References

1. Heinaniemi M, Vuorenmaa T, Teppo S, Kaikkonen MU, Bouvy-Liivrand M, Mehtonen J, et al. Transcription-coupled genetic instability marks acute lymphoblastic leukemia structural variation hotspots. Elife. 2016;5.
2. Steidl C, Diepstra A, Lee T, Chan FC, Farinha P, Tan K, et al. Gene expression profiling of microdissected Hodgkin Reed-Sternberg cells correlates with treatment outcome in classical Hodgkin lymphoma. Blood. 2012;120(17):3530-40.
3. Kim D, Pertea G, Trapnell C, Pimentel H, Kelley R, Salzberg SL. TopHat2: accurate alignment of transcriptomes in the presence of insertions, deletions and gene fusions. Genome Biol. 2013;14(4):R36.
4. Li H, Handsaker B, Wysoker A, Fennell T, Ruan J, Homer N, et al. The Sequence Alignment/Map format and SAMtools. Bioinformatics. 2009;25(16):2078-9.
5. Quinlan AR, Hall IM. BEDTools: a flexible suite of utilities for comparing genomic features. Bioinformatics. 2010;26(6):841-2.
6. Trapnell C, Roberts A, Goff L, Pertea G, Kim D, Kelley DR, et al. Differential gene and transcript expression analysis of RNA-seq experiments with TopHat and Cufflinks. Nat Protoc. 2012;7(3):562-78.
7. R-Development-Core-Team. R: A language and environment for statistical computing.: R Foundation for Statistical Computing, Vienna, Austria.; 2008 [Available from: http://www.R-project.org.
8. Kim D, Langmead B, Salzberg SL. HISAT: a fast spliced aligner with low memory requirements. Nat Methods. 2015;12(4):357-60.
9. Love MI, Huber W, Anders S. Moderated estimation of fold change and dispersion for RNA-seq data with DESeq2. Genome Biol. 2014;15(12):550.
10. Liao Y, Smyth GK, Shi W. featureCounts: an efficient general purpose program for assigning sequence reads to genomic features. Bioinformatics. 2014;30(7):923-30.
11. Huang da W, Sherman BT, Lempicki RA. Systematic and integrative analysis of large gene lists using DAVID bioinformatics resources. Nat Protoc. 2009;4(1):44-57.
12. Huang da W, Sherman BT, Lempicki RA. Bioinformatics enrichment tools: paths toward the comprehensive functional analysis of large gene lists. Nucleic Acids Res. 2009;37(1):1-13.
13. Bindea G, Mlecnik B, Hackl H, Charoentong P, Tosolini M, Kirilovsky A, et al. ClueGO: a Cytoscape plug-in to decipher functionally grouped gene ontology and pathway annotation networks. Bioinformatics. 2009;25(8):1091-3.
14. Bindea G, Galon J, Mlecnik B. CluePedia Cytoscape plugin: pathway insights using integrated experimental and in silico data. Bioinformatics. 2013;29(5):661-3.
15. Shannon P, Markiel A, Ozier O, Baliga NS, Wang JT, Ramage D, et al. Cytoscape: a software environment for integrated models of biomolecular interaction networks. Genome Res. 2003;13(11):2498-504.
16. Langmead B, Salzberg SL. Fast gapped-read alignment with Bowtie 2. Nature methods. 2012;9(4):357-9.
17. Zhu LJ, Gazin C, Lawson ND, Pages H, Lin SM, Lapointe DS, et al. ChIPpeakAnno: a Bioconductor package to annotate ChIP-seq and ChIP-chip data. BMC Bioinformatics. 2010;11:237.
18. Saldanha AJ. Java Treeview--extensible visualization of microarray data. Bioinformatics. 2004;20(17):3246-8.
19. Eijssen LM, Jaillard M, Adriaens ME, Gaj S, de Groot PJ, Muller M, et al. User-friendly solutions for microarray quality control and pre-processing on ArrayAnalysis.org. Nucleic Acids Res. 2013;41(Web Server issue):W71-6.
20. Buenrostro JD, Wu B, Chang HY, Greenleaf WJ. ATAC-seq: A Method for Assaying Chromatin Accessibility Genome-Wide. Curr Protoc Mol Biol. 2015;109:21 9 1-9.
21. Corces MR, Buenrostro JD, Wu B, Greenside PG, Chan SM, Koenig JL, et al. Lineage-specific and single-cell chromatin accessibility charts human hematopoiesis and leukemia evolution. Nat Genet. 2016;48(10):1193-203.

3. Supplementary Tables

Table 1. qPCR gene expression primers. (*) sequences obtained from PrimerBank.

Primer	Genomic Co-ordinates (hg19)	Forward	Reverse
CSF1R-LTR	Chr5:149472147- 149472167<br>Chr5:149460507 – 149460526	TTGGATGTGATTCTGCTCCTC	CCACACATCGCAAGGTCAC
CSF1R EX2/3	Chr5: 149465998-149466015<br>Chr5: 149460507-149460525	CACCTGCCTGCCACTTCC	CCACACATCGCAAGGTCAC
NLRP1 LTR	Chr17: 5487775- 5487794<br>Chr17: 5522717- 5522736	TTCAGACCTCTCCAGGCCCT	TCTCCTGCCGCCATTTGAAG
NLRP1	Chr17: 5487566- 5487586<br>Chr17: 5522778- 5522800	CGTCACCACATCTCCCCTCAC	CAGATCTCGTGGGAACTCACTCA
CACNA2D1 Ex2 > LTR	Chr7: 81978946- 81978965<br>Chr7: 81964509- 81964529	TATCAAATCATGGGTGGATA	ACCAGCTGGCGTGCATTATTT
CACNA2D1 LTR > Ex5	Chr7: 81765951- 81765968<br>Chr7: 81962766- 81962786	TTGCTCCTCCTTTGCCTTCCG	ATCGAGATCATCCTTTGC
CHD1L-AS	Chr1: 146786722- 146786741<br>Chr1: 146767241- 146767261	CCATGTGAGATGTGTCTTTC	CAGAGGTACTGCAATAGAGTA
lncRNA	Chr6: 81662281- 81662301<br>Chr6:81662248-81662268	CCTTCAACTTCAGCCATGATT	ACTCACAGTTCAGCATGGCT
CBFA2T3	Chr16: 88958698- 88958717<br>Chr16: 88958333- 88958352	CAGTTTGGCAGCGACATCTC	GCCTCCTGAAGCTTGGAATG
GAPDH	Chr12: 6647093- 6647112<br>Chr12: 6647279- 6647299	CCCACTCCTCCACCTTTGAC	ACCCTGTTGCTGTAGCCAAAT
TNFRSF11A *(*****22547111c1)*	Chr18: 60015411- 60015431<br>Chr18: 623499236/2354392-62354397	AGATCGCTCCTCCATGTACCA	GCCTTGCCTGTATCACAAACTTT
TNFRSF11A LTR	Chr18:59990862-59990883<br>Chr18: 60015413-60015433	AGCCACATGGAACTGTAAGTC	ACTGGTACATGGAGGAGCGA
WNT5A *(*****371506361c1)*	Chr3: 55514821/ 55513559-55513570<br>Chr3: 55513444- 55513463	ATTCTTGGTGGT/CGCTAGGTA	CGCCTTCTCCGATGTACTGC
WNT5A LTR	Chr3: 55539260- 55539278<br>Chr3: 55514867 -55514885	GCTGCCACCTTGTGAAGAA	GCCACTAGGAAGAACTTGG
WNT5A LTR-Flanking	Chr3: 55539203 - 55539220<br>Chr3: 55539003 - 55539024	TTTCCTGAGGCCTCCTGA	CTTTCACCCAGTAGGCTGTAAG

Table 2. siRNA

Target	siRNA	Manufacturer
**WNT5A**	sasi_hs01_00200618	Sigma Aldrich
**TNFRSF11A**	sasi_hs01_00186225	Sigma Aldrich
**Non-targeting control**	non-targeting control pool #2	Dharmacon

Table 3. Western blotting antibodies.

Antibody	Dilution
NF-κB p65 (6956s - Cell Signalling)	1:1,000
β-Actin (A1978 – Sigma)	1:5,000
Anti-Mouse HRP (Jackson ImmunoResearch)	1:10,000
Anti-Rabbit HRP (Jackson ImmunoResearch)	1:10,000
RANK (TNFRSF11A) (ab13918 - Abcam)	1:5,000
WNT5 A/B (2530S – Cell Signalling)	1:5,000
GAPDH (6C5) (ab8245 – Abcam)	1:10,000

Table 4. Alignment rates of RACE-Seq experiments

Sample	Total reads	Aligned reads
KM-H2_1	8,392,316	7,669,431
KM-H2_2	15,174,978	13,580,848
KM-H2_3	14,064,652	13,185,031
L1236_1	6,907,526	6,218,993
L1236_2	24,749,368	18,780,381
L1236_3	41,434,210	34,779,746
L428_1	7,742,210	7,075,672
L428_2	17,384,734	9,781,685
L428_3	41,607,680	28,985,146
Namalwa_1	11,121,412	7,943,909
Namalwa_2	7,726,574	7,004,466
Namalwa_3	25,769,872	18,735,154
Reh_1	7,137,592	5,230,253
Reh_2	8,906,268	3,441,643
Reh_3	18,962,882	11,471,231
Reh_PMA_1	11,513,294	8,313,568
Reh_PMA_2	14,527,438	11,681,652
Reh_PMA_3	11,862,250	10,036,056
Reh_GFP_plus_Dox_1	7,949,198	1,292,269
Reh_GFP_plus_Dox_2	24,827,202	3,868,880
Reh_GFP_plus_Dox_3	7,983,918	777,684
Reh_IKK_no_Dox_1	9,418,400	1,136,992
Reh_IKK_no_Dox_2	11,485,612	1,452,205
Reh_IKK_no_Dox_3	6,733,262	1,160,301
Reh_IKK_plus_Dox_1	7,670,960	1,838,776
Reh_IKK_plus_Dox_2	6,790,874	929,711
Reh_IKK_plus_Dox_3	8,585,052	1,636,404

Table 5. Alignment rates of cell line RNA-Seq experiments

Sample	Total reads	Aligned reads
KM-H2_1	45,849,821	40,089,597
KM-H2_2	24,039,892	22,124,324
L1236_1	59,405,615	52,405,097
L1236_2	122,922,885	109,057,912
L428_1	76,682,880	68,746,335
L428_2	80,351,196	78,219,910
Namalwa_1	41,690,209	37,122,799
Namalwa_2	31,491,810	29,422,730
Reh_1	93,895,915	83,741,217
Reh_2	34,685,724	33,013,221
Reh_PMA_1	78,489,893	75,712,210
Reh_PMA_2	72,645,208	69,090,152

Table 6. Alignment rates of LCM RNA-Seq experiments

Sample	Total reads	Aligned reads
LCM1_NTC	63,628,519	52,955,654
LCM1_TU	71,764,441	65,308,883
LCM2_NTC	60,377,862	36,121,729
LCM2_TU	121,177,416	80,341,068
LCM3_NTC	66,031,555	47,710,910
LCM3_TU	80,648,430	70,807,319
LCM4_NTC	36,422,883	28,053,424
LCM4_TU	55,256,148	26,144,587
LCM5_NTC	65,698,071	37,978,976
LCM5_TU	75,497,798	56,567,598
